# Efficacy and safety of mesenchymal stem/stromal cells and their derived extracellular vesicles for acute respiratory distress syndrome: a systematic review and meta-analysis

**DOI:** 10.1186/s13287-025-04644-4

**Published:** 2025-09-29

**Authors:** Yaxin Wu, Ruonan Xu, Yuanyuan Li, Kai Liu, Tao Yang, Ming Shi, Fu-Sheng Wang, Zhe Xu

**Affiliations:** 1 Senior Department of Infectious Diseases, Chinese PLA General Hospital, National Clinical Research Center for Infectious Diseases, Beijing, China; 2 The First Affiliated Hospital of USTC, Division of Life Sciences and Medicine, University of Science and Technology of China, Hefei, China

**Keywords:** Mesenchymal stem cells, Extracellular vesicles, Efficacy, Safety, All-cause mortality

## Abstract

**Background:**

Although numerous clinical trials have explored stem cell-based therapies for acute respiratory distress syndrome (ARDS), their findings are inconsistent. This meta-analysis aimed to comprehensively evaluate the efficacy and safety of stem cell-based therapies, including mesenchymal stem/stromal cells (MSCs) and their derived extracellular vesicles (EVs), in the treatment of ARDS.

**Methods:**

A comprehensive literature search of the Cochrane Library, PubMed, and Web of Science databases and the US National Institutes of Health Trials Registry (ClinicalTrials.gov) was conducted to identify eligible studies assessing the efficacy and safety of stem cell-based therapies in ARDS. The primary outcomes included all-cause mortality within or over one month, adverse events (AEs), and serious adverse events (SAEs). To explore possible bias, subgroup analysis was performed based on the design of study (randomized controlled trial vs. nonrandomized interventional trial), etiology of ARDS, type of stem cell-based therapy, and times of infusion. Relative risk (RR) and mean difference (MD) were calculated to evaluate efficacy and safety. This study was registered with PROSPERO (CRD42024593740).

**Results:**

A total of 48 studies involving 1,773 patients were eligible, of which 31 studies were included in the meta-analysis. The results revealed a significant reduction in all-cause mortality among patients receiving MSCs or their derived EVs and secretomes compared to those receiving routine therapy (RR = 0.74, 95% CI = 0.63–0.87, *p* = 0.0003, *I²*=5%). This effect was only seen in all-cause mortality within one month (RR = 0.74, 95% CI = 0.62–0.89, *p* = 0.002, *I²*=0%); furthermore, high dose MSCs (over 1 × 10^6^ cells/kg or 7 × 10^7^ cells per infusion) was associated with reduction of all-cause mortality in ARDS (RR = 0.70, 95% CI = 0.55–0.89). There were no significant differences in AE (RR = 1.08, 95% CI = 0.97–1.21, *p* = 0.17, *I*^*2*^ = 26%) or SAE (RR = 0.94, 95% CI = 0.80–1.11, *p* = 0.49, *I*^*2*^ = 0) between the stem cell-based therapy group and the control group. In addition, MSC-derived EVs and secretomes demonstrated preliminary efficacy in the treatment of ARDS (RR = 0.63, 95% CI = 0.46–0.86, *p* = 0.003, *I*^*2*^ = 40%).

**Conclusions:**

Stem cell-based therapy significantly reduced mortality within one month and was well tolerated in ARDS patients. Given the limited sample size of included studies, the efficacy of stem cell-based therapy in patients with ARDS needs to be validated in further larger and more rigorous randomized controlled trials.

**Supplementary Information:**

The online version contains supplementary material available at 10.1186/s13287-025-04644-4.

## Introduction

Acute respiratory distress syndrome (ARDS) is characterized by the acute onset of hypoxemia and bilateral pulmonary edema resulting from excessive alveolocapillary permeability, leading to high morbidity and mortality rates [1], which presents in 10% of the intensive care units (ICUs) patients and 23% of those on mechanical ventilated patients, with hospital mortality rates ranging from 35 to 45% [2, 3]. For patients in ICU, ARDS was found to increase the mortality rate by 15%, and severe ARDS increase by 23% [4]. Reports from 18 ICUs in mainland China indicates an even higher mortality rate of 46.3%, which rose to 56.2% in cases of severe ARDS [5]. However, current ARDS management strategies focus on symptomatic relief and disease progression prevention through respiratory support, mechanical ventilation, and corticosteroids. These approaches have limited efficacy in addressing the underlying airway damage, cytokine storm, and other harmful effects caused by the inflammatory response [6, 7]. As such, there is an urgent need for innovative therapies that target severe respiratory function disorders.

In this context, stem cell-based therapies, including stem cells, their derived extracellular vesicles (EVs), and secretomes, have emerged as promising adjunctive treatments on ARDS. Mesenchymal stem cells (MSCs) can be sourced from various autologous and allogeneic tissues, including bone marrow, adipose tissue, umbilical cord, Wharton’s jelly, peripheral blood, menstrual blood, et al. [8–10]. EVs was secreted by MSCs, carrying proteins, miRNA, and mRNA species that influence signaling responses in target cells to modulate inflammatory and repair responses. Their unique properties-such as pluripotent differentiation, low immunogenicity, and immunomodulatory capabilities—position MSC-based therapies as promising adjunctive treatments for ARDS [11–13].

Numerous clinical trials and meta-analyses have demonstrated the efficacy of MSCs in hospitalized and severe COVID-19 patients [14–17]. However, REALIST-COVID trial found no improvement in pulmonary organ dysfunction after receiving MSCs and even suggested an association between MSCs infusion and prolonged ventilation [18]. In contrast, Grégoire et al. [19] reported a reduction in mortality among ARDS patients receiving MSCs [19].

Recent years, emerging research has highlighted the potential benefits of MSC-derived extracellular vesicles (EVs) in the treatment of severe COVID-19 preliminary [20, 21], representing an important step forward in the clinical translation of MSC-derived EVs. Previous meta-analyses have established that MSCs can reduce ARDS mortality without increasing adverse events (AEs) [22, 23]. However, these meta-analyses included a limited number of studies, did not account for the latest research involving EVs, and only assessed the impact of MSCs on mortality without considering other outcome measures.

To address these evidence gaps and update previous meta-analyses, we conducted a comprehensive meta-analysis of randomized controlled trials (RCTs) and non-randomized interventional trials (NRITs) to evaluate the efficacy and safety of stem cell-based therapy for ARDS.

## Methods

This systematic review and updated meta-analysis followed the Preferred Reporting Items for Systematic Reviews and Meta-Analysis (PRISMA) guidelines [24] (Additional Table S1). Furthermore, it is registered on the International Prospective Register of Systematic Reviews (PROSPERO) website (CRD42024593740).

### Search strategy

A comprehensive literature search was conducted across the Cochrane Library, PubMed, Web of Science databases, and the US National Institutes of Health Trials Registry (ClinicalTrials.gov). The following medical subject headings and free terms were used: mesenchymal stem cells, mesenchymal stromal cells, extracellular vesicle, ARDS, and COVID-19. In addition, all references from the identified reviews and selected articles were screened to find additional relevant studies. The search was limited to English journal articles, but no limit was placed on the publication date. The detailed search strategy can be found in additional Table S2.

### Inclusion criteria

The eligible studies for this meta-analysis were identified using the population, intervention, comparison, outcomes, and study design (PICOS) principles. The following inclusion criteria were used:

- Population (P): Studies of patients with ARDS (PaO_2_/FiO_2_ ratio ≤ 300 mmHg), regardless of the cause.
- Intervention (I): Studies focused on cell therapy transplants that included stem cells, MSCs, mesenchymal stromal cells, medicinal stem cells, induced pluripotent stem (iPS) cells, induced pluripotent stem cells (iPSC), progenitor cells, and/or EVs.
- Comparison (C): Studies with or without control group received the standard treatment.
- Outcomes (O): Studies that measured mortality, AEs, serious adverse events (SAEs), ventilator-free days, duration of ventilation, ICU-free days, length of ICU stay, days of hospitalization.
- Study design (S): Interventional studies.

### Exclusion criteria

Conference abstracts or letters with duplicates, meta-analyses, reviews, editorials, studies in vitro, animal studies, cost-benefit analyses, and studies with incomplete or unavailable data were excluded.

### Study selection

Of the 6065 studies initially retrieved from the literature, 5163 potentially relevant articles were screened after duplicates were removed. After screening the titles and abstracts, the full texts of the remaining 71 articles were retrieved for further screening. Disagreements during the screening process were resolved through discussion with a third member.

### Data extraction

Two reviewers independently extracted the following information from the selected studies: title, author(s), year, location, design of study, inclusion and exclusion criteria of patients, concomitant medications for ARDS (e.g., Dexamethason, Heparin, Lopinavir-Ritonavir, Remedesivir, et al.), coexisting illnesses, baseline cytokine levels, e.g., Interleukin-6 (IL-6), c-reactive protein (CRP), sample size, age, gender, follow-up time, MSC source, times and dose of MSCs, mortality, ventilator-free days, duration of ventilation, ICU-free days, length of ICU stay, days of hospitalization, AEs, SAEs, laboratory test (CRP, ferritin, D-dimer, IL-6, TNF-α, etc.), and PaO_2_/FiO_2_ ratio. A standardized data extraction form in Microsoft Excel was used to collect this information.

### Data transformation

For the continuous outcome variables (e.g., ventilator-free days, duration of ventilation, ICU-free days within one month, length of ICU stay, days of hospitalization), mean and standard deviation (SD) values for the stem cell-based therapy and control groups were extracted from each of the selected studies. When SDs for a group of means were not provided, they were calculated from the standard error of the mean (SEM) or 95% confidence intervals (CIs) using the following equations from Chap. 6.5.2.2 of the Cochrane Handbook: [$\:SD=SEM\times\:\sqrt{n}]$ or $\:[SD=\sqrt{n}\times\:(upper\:limit-lower\:limit)/\text{3,92}$]. Parameters provided in medians and the 25th–75th percentile were converted into means ± SD using Wan et al.’s equation in Chap. 6.5.2.5 of the Cochrane Handbook [25].

### Quality assessment and bias exploration

To assess the risk of bias, we used the Cochrane Collaboration’s Risk of Bias 1 tool for the RCTs [26], the ROBINS-I V2 tool for the NRITs [27, 28], and the Joanna Briggs Institute (JBI) methodology checklist for case report and case series [29]. The heterogeneity of the included studies was assessed based on a Q-test and quantified with *I*^*2*^ [30, 31]. We assessed possible publication bias by testing for asymmetry using a funnel plot. If possible publication bias was present, we used Egger’s regression intercept test to evaluate publication bias across studies [32].

### Outcome measures

The primary efficacy outcome was all-cause mortality. The additional efficacy outcome included ventilator-free days, duration of ventilation, ICU-free days within one month, length of ICU stay, and days of hospitalization. The primary safety outcomes were AE and SAE.

### Data analysis

Relative risk (RR) with a 95% CI and mean difference (MD) were used to estimate the efficacy and safety of the intervention for dichotomous outcomes and continuous outcomes, respectively. The pooled effect size was calculated using either the Mantel-Haenszel method (fixed-effects model) or the Der Simonian-Laird method (random-effects model). If *I*^*2*^ ≤ 50%, then the homogeneity between studies was mild, and the fixed-effects model was used. To explore the potential for bias, a subgroup analysis was conducted based on the design of study, the cause and categories of ARDS, type of stem cell-based therapies (MSCs or EVs), the times of infusion, dose (over 1 × 10^6^ cells/kg or 7 × 10^7^ cells per infusion was considered high dose) and mortality within or over one month. A sensitivity analysis was carried out to evaluate the stability of the pooled results by removing each of the included studies. Publication bias was determined using a funnel plot.

In addition, since there were single-arme studies, case reports, case series studies, we identified efficacy outcomes in these studies that were reported inconsistently or infrequently and were more suitable for a narrative analysis than a meta-analysis, mainly including the change of CT imaging, laboratory test, clinical treatment outcome (safety and efficacy).

Review Manager (RevMan) version 5.3 (Nordic Cochrane Centre, Copenhagen, Denmark) and RStudio version 4.4.1 were used to perform all statistical analyses. Statistical significance was set at *p* < 0.05.

## Results

### Study characteristics

**Fig. 1:**
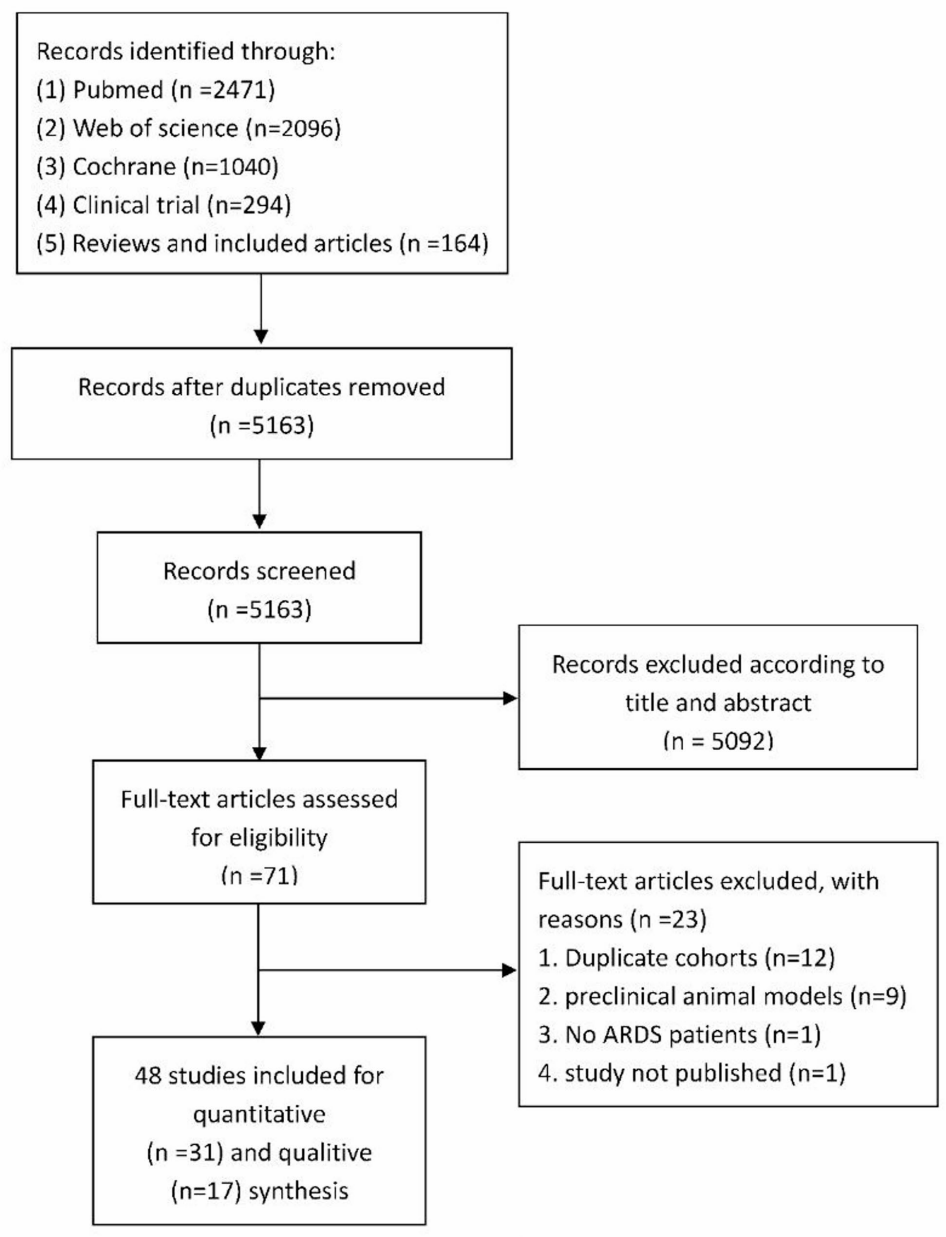
Flow diagram for the search and selection of eligible studies

A total of 48 studies (18 NRITs, 24 RCTs, 3 case report, and 3 case series), involving 1,773 patients, met the inclusion criteria and were included in the systematic review and meta-analysis [14–18, 20, 21, 33–73] (31 for quantitative analysis and 17 studies for qualitive synthesis). The detailed search results can be found in Fig. 1. Of these studies, 38 focused on COVID-19-related ARDS and 8 focused on pneumonia or sepsis-related ARDS, 1 for HIN1, and 1 for H7N9. Male patients accounted for 60% of the patients included.

The infusion dose was determined based on patient weight (5 × 10^5^ ~ 10 × 10^6^ cells/kg) or a fixed dose(3 × 10^7^ ~ 9 × 10^8^ cells). Of the selected studies, 20 assessed the efficacy and safety of umbilical cord-derived MSCs (UC-MSCs) [15, 16, 18, 34–42, 58, 62–68], 8 assessed bone marrow-derived MSCs (BM-MSCs) [14, 43–45, 57, 59–61], 4 assessed WJ-MSCs [46, 47, 69, 70], 4 assessed AD-MSCs [48–51], 6 assessed EVs or secretomes [20, 21, 33, 71–73], 2 assessed menstrual blood-derived MSCs (Men-MSCs) [52, 53], 2 assessed multipotent adult progenitor cells [17, 54], 2 assessed placenta-derived MSCs (PL-MSCs) [55, 66], and 1 assessed MSCs plus EVs [56]. Table 1 shows the detailed characteristics of the included studies, including design of study, age, gender, dose, and times of infusion.

**Table 1:** Characteristics of included studies

Study	Location	Phase	Study design	Causes of ARDS	Sample size (treatment; control)	Age (years) (treatment; control)	Male (%) (treatment; control)	MSCs or EVs source	times	Dose per infusion
Zheng-2014 [48]	China	Phase 1	RCT	Pneumonia	6; 6	Mean (SD): 66.7 (20.4); 69.8 (9.1)	100; 83.3	AD	Single	1 × 10^6^ cells/kg
Matthay-2019 [14]	USA	Phase 2a	RCT	Sepsis, pneumonia	40; 20	Mean (SD): 55 (17); 55 (20)	58.0; 50.0	BM	Single	1 × 10^6^ cells/kg
Lanzoni-2021 [42]	USA	Phase 1/2a	RCT	COVID-19	12; 12	Mean (SD): 58.58 (15.93); 58.83 (11.61)	41.7; 66.7	UC	Two	100 ± 20 × 10^6^ cells
Dilogo-2021 [16]	Indonesia	Phase 1	RCT	COVID-19	20; 20	-	75.0; 75.0	UC	Single	1 × 10^6^cells/kg
Shi-2021 [41]	China	Phase 2	RCT	COVID-19	65; 35	Mean (SD): 60.72 (9.14); 59.94 (7.79)	56.9; 54.3	UC	Three	4 × 10^7^ cells
Adas-2021 [47]	Turkey	Phase 1/2	RCT	COVID-19	10; 10	-	-	WJ	Three	3 × 10^6^ cell/kg
Monsel-2022 [40]	France	Phase 2b	RCT	COVID-19	21; 24	Mean (SD): 64 (10.4); 63.2 (11.4)	81.0; 83.3	UC	Three	1 × 10^6^ cells/kg
Rebelatto-2022 [15]	Brazil	Phase 1/2	RCT	COVID-19	11; 6	Mean (SD): 53(15.3); 61.7(9.7)	72.7; 66.6	UC	Three	5 × 10^5^ cells/kg
Aghayan-2022 [56]	Iran	Phase 1	RCT	COVID-19	10; 10	Mean: 62.3; 58.4	-	PL	Single	1 × 10^6^ cells/kg
Shu-2020 [39]	China	Phase 1	RCT	COVID-19	12; 29	Mean (SD): 61.00 (17.87); 57.86 (15.79)	66.7; 55.2	UC	Single	2 × 10^6^ cells/kg
Bowdish-2022 [43]	USA	Phase2/3	RCT	COVID-19	112; 110	Mean (SD): 61.8 (13.0); 59.6 (13.8)	70.5; 68.2	BM	Two	2 × 10^6^ cells/kg
Kaffash-2022 [38]	Iran	Phase 1	RCT	COVID-19	10; 10	Mean (SD): 62.00(2.42); 61.3(5.34)	70.0; 60.0	UC	Three	1 × 10^6^ cells/kg
Leng-2020 [49]	China	Polit study	NRIT	COVID-19	7; 3	-	57.1; 0	AD	Single	1 × 10^6^ cells/kg
Meng-2020 (37]	China	Phase 1	NRIT	COVID-19	9; 9	-	77.8; 44.4	UC	Three	3 × 10^7^ cells
Xu-2021 [53]	China	Phase 1	NRIT	COVID-19	26; 18	Mean (SD): 58.31(12.49); 61.11(11.03)	65.4; 72.2	menstrual blood	Three	9 × 10^7^ cells
Wei-2021 [34]	China	-	NRIT	COVID-19	13; 12	Median (IQR): 67 (56–70);	58.3; 38.5	UC	Single	1 × 10^6^ cells/kg
Chen-2020 [52]	China	phase 1/2	NRIT	H7N9	17; 44	Mean (SD): 62.8 (14.4); 61.6 (11.8)	-	menstrual blood	Three/four	1 × 10^6^ cells/kg
Bukreieva-2023 [36]	Ukraine	phase 1/2	NRIT	COVID-19	13; 15	Median (range): 58 (32–71); 62 (32–73)	61.5; 73.3	UC	Three	1 × 10^6^ cells/kg
Zarrabi-2023 [57]	Iran	phase 2	RCT	COVID-19	MSCs group (*n* = 11) MSCs + EVs group (*n* = 8); 24	MSCs group:50 ± 12.48; MSCs + EVs group:47.75 ± 12.72; 49.4 (11.87)	MSCs group: 90.9, MSCs + EVs group: 62.5; 66.7	MSCs/MSCs + EVs	Two	100 × 10^6^ cells
Ichikado-2023 [54]	Japan	phase 2	RCT	Pneumonia	19; 7	Mean (SD): 69.2 (13.2); 66.5 (10.8)	80.0; 100.0	MAPC	Single	9.0 × 10^8^ cells
Bellingan-2022 [55]	UK	phase 1/2	RCT	Sepsis, pneumonia	20; 10	Mean (SD): 51 (14); 59 (18)	65.0; 60.0	MAPC	Single	9.0 × 10^8^ cells
Gorman-2023 [18]	UK	Phase 2	RCT	COVID-19	30; 29	Mean (SD): 58.4 (9.2); 58.4 (12.5)	80.0; 69.0	UC	Single	400 × 10^6^ cells
Pochon-2023 [46]	France	Phase 2a	RCT	COVID-19	15; 15	Median (IQR): 61 (49–66); 66 (61–70)	87.0; 47.0	WJ	Three	1 × 10^6^ or 0.5 × 10^6^ cells/kg
Grégoire-2022 [44]	Belgium	Phase 1/2	NRIT	COVID-19	8; 24	Median (IQR): 50 (43–58); 54 (49.5–63)	87.5; -	BM	Three	1.5-3 × 10^6^ cells/kg
Simonson-2015 [58]	multicenter	-	Case report	H1N1, chemotherapy-induced neutropenia	2	Range: 29–86	22.2	BM	Single	2 × 10⁶ cells/kg
Chen-2022 [59]	Taiwan, China	-	Retrospective case-control study	COVID-19	21	Median 51.00 (19.00–62.00)	61.9	UC	Single or two	1.0 × 10⁸ cells
Haberle-2021 [60]	USA	-	Retrospective comparative study	COVID-19	23	Mean (IQR): 39 (32–50); 59 (54–79)	60; 72.2	BM	Single	1 × 10⁶ cells/kg
Brown-2022 [61]	UK	-	Case series	COVID-19	11	Median (IQR): 51 (39–60)	90.9	BM	Single	1 × 10⁶ cells/kg
Wilson-2015 [62]	USA	Phase 1	Open-label, dose-escalation trial	Preeclampsia, pneumonia, aspiration, sepsis	9	Range: 29–86	22.2	BM	Single	1, 5, 10 × 10⁶ cells/kg
Yip-2020 [63]	Taiwan, China	Phase 1	Prospective clinical trial	Pneumonia	9	Mean (SD): 54 ± 18	77.8	UC	Single	1, 5, 10 × 10⁶ cells/kg
Gorman-2021 [64]	UK	Phase 1	Open-label, dose-escalation trial	Gastric content aspiration, thoracic trauma, pneumonia, sepsis	9	Range: 25–83	66.7	UC	Single	2 × 10⁶ cells/kg
Feng-2021 [65]	China	pilot trial	Single arm	COVID-19	16	Mean (SD): 61.75 (10.02)	75.0	UC	Four	1 × 10⁸ cells
Tao-2020 [66]	China	-	Case report	COVID-19	1	72	100	UC	Five	1.5 × 10⁶ cells/kg
Hashemian-2021 [67]	Iran	-	Case series	COVID-19	11	Mean (SD): 53.8 (10.37)	72.7	UC or PL	Three	200 × 10⁶ cells
Guo-2020 [68]	China	-	Case series	COVID-19	31	Median (IQR): 70 (61–71)	80.6	UC	-	-
Ercelen-2021 [69]	India	-	Single arm	COVID-19	210	Mean (SD): 61.22 (11.77); 57.36 (12.00)	72.9	UC	Single	2 × 10⁷ cells
Zhang-2020 [70]	China	-	Case report	COVID-19	1	54	100	WJ	Single	1 × 10⁶ cells/kgs
Saleh-2021 [71]	Iran	Phase 1	NRIT	COVID-19	5	Range: 45–54	60.0	WJ	Three	150 × 10⁶ cells
Zamanian-2024 [21]	Iran	Phase 2/3	RCT	COVID-19	21;21	Mean (SD): 54.24(15.93); 62.08(16.66(	81.0;81.0	PL (EVs)	Two	1.5-2 × 10^9^ EVs/kg
Lightner-2023 [20]	USA	Phase 2	RCT	COVID-19	34, 34; 34	Mean (SD): ExoFlo 15 mL: 56.8 (14.97); ExoFlo 10 mL:62.1 (13.47); 58.5 (11.76)	54.5, 61.9; 41.7	BM (EVs)	Two	1.2 or 0.9 × 10^12^ EVs
Stewart-2023 [35]	Canada	Phase 2	RCT	COVID-19	14; 8	-	-	UC	Three	90 × 10^6^ cells
Martínez-Muñoz-2024 [45]	Spain	NR	RCT	COVID-19	10; 10	Median (range): 59.5 (47–77); 65.5 (46–75)	50.0; 80.0	BM	Single	0.99 ± 0.06 × 10^6^/kg
Laterre-2024 [50]	multicenter	Phase 1b/2a	RCT	Severe CABP	42; 41	Mean (SD): 61.1 (11.2); 63.4 (10.4)	67.0; 64.0	AD	Two	1.6 × 10^8^ cells
Fathi-Kazerooni-2022 [33]	Iran	Phase 1/2	RCT	COVID-19	14; 15	Mean (SD): 46.43 (11.91); 53.67 (10.30)	60.0; 67.0	menstrual blood (EVs)	Five	5 mL secretome
Zhu-2022 [72]	USA	Phase 2a	Single arm	COVID-19	7	Median (IQR): 57 (43–70)	57.1	AD (EVs)	Five	2–6 × 10^8^ particles
Sengupta − 2020 [73]	USA	-	NRIT	COVID-19	27	Median (range): 59 (29–84)	63.0	BM (EVs)	Single	15 ml
Chu-2022 [74]	China	Pilot trial	Single arm	COVID-19	7	Range: 19–62	57.1	UC (EVs)	Twice a day	7.66e + 0.8 to 7.00e + 0.7 particles/ml
de Dios-2023 [51]	USA	Phase 2	RCT	COVID-19	33; 15	Mean (SD): 53.2 (17.6); 54.7 (15.8)	54.5.0; 66.7.0	AD	Four	1 × 10^8^ cells

SD: standard deviation; IQR: Inter Quartile Range; UC: umbilical cord; WJ: Wharton Jelly; EVs: extracellular vesicles; PL: placenta; BM: bone-marrow; AD: adipose; CABP: community-acquired bacterial pneumonia; -: not reported. MAPC: multipotent adult progenitor cells;

Table S7 covers coexisting illnesses, concomitant medications, and baseline cytokine levels. Hypertension and diabetes were the most common coexisting illnesses. Nearly half of the patients were diagnosed with hypertension (stem cell-based therapy group: 43.1%; control group: 47.4%), as reported by 26 studies; nearly one-third of the patients had diabetes (stem cell-based therapy group: 26.1%; control group: 27.2%), as reported by 27 studies. In addition, common concomitant medication mainly includes vasopressors, vasopressors and corticosteroids. Notably, 64.5% of the stem cell-based therapy group and 73.8% of the control group received corticosteroids during hospital stays. The baseline serum CRP and IL-6 values varied from normal to even more than 100 times the upper limit of normal (ULN).

### Risk of bias

Of the RCTs, five were at high risk of performance bias, five of detection bias, three of selection bias, four of attrition bias, and two of reporting bias. Of the NRITs, fifteen were at moderate risk because of moderate risk bias due to deviations from intended interventions. Additionally, 3 case series studies and 3 case-control studies complied with all JBI criteria for good-quality studies. Among ten cohort studies, methodological ambiguities were noted: eight had unclear details on outcomes or exposure measurements, and two lacked clarity regarding sample sources. Regarding on case series studies, one enrolled non-consecutive patients, while two recruited participants from a single center. Additionally, two case series studies did not evaluate the potential influence of geographic or sociological factors. One case series study reported inadequate baseline clinical information. The details of the assessment are listed in Additional Tables S3, S4, S5 and S6.

### Meta-analysis of all-cause mortality

**Fig. 2:**
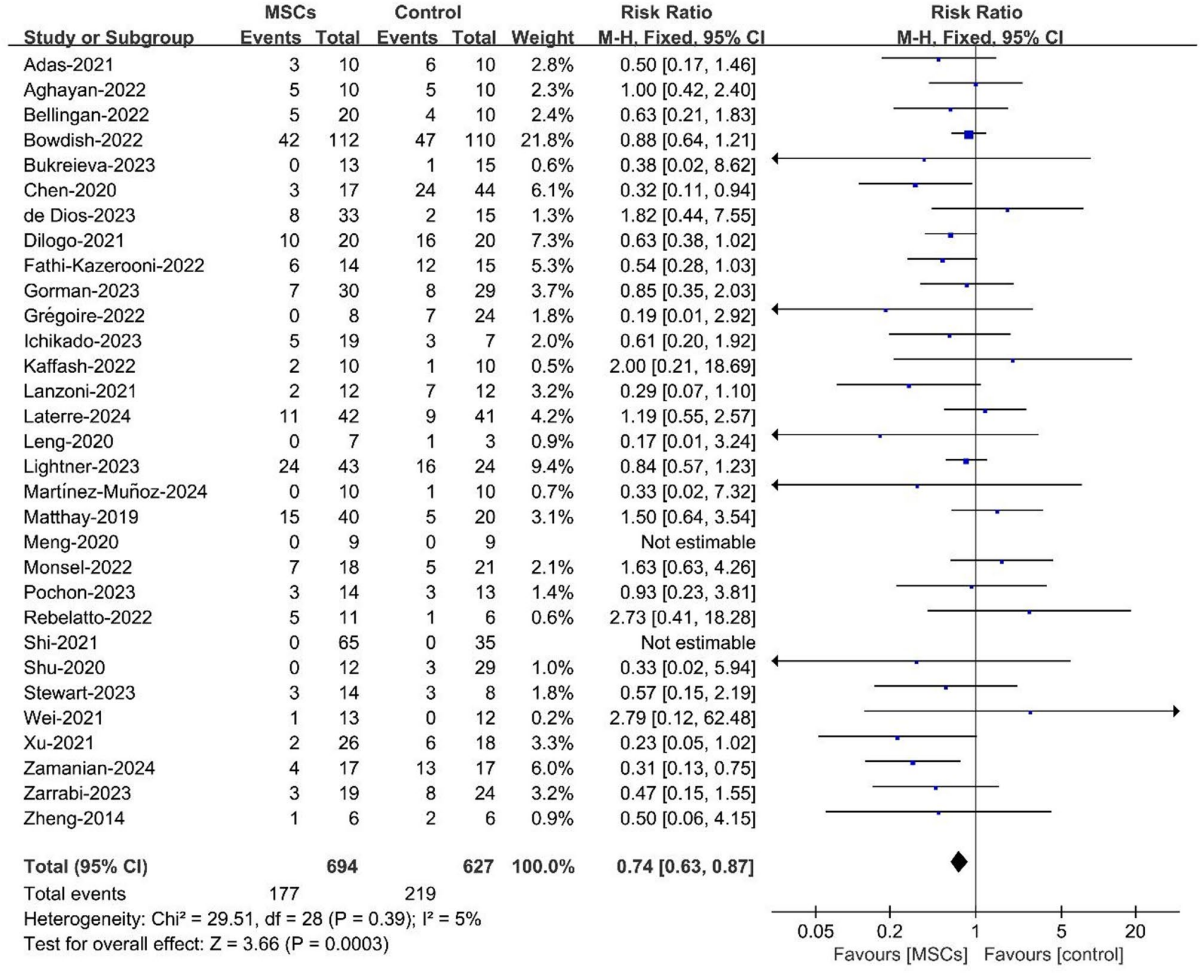
Forest plot of the pooled results for all-cause mortality

The 31 selected studies reported on all-cause mortality after stem cell-based therapy in 1,321 ARDS patients. Compared with the standard therapy, MSCs significantly reduced the primary efficacy endpoint of all-cause mortality (RR = 0.74, 95% CI = 0.63–0.87, *p* = 0.0003, *I²*=5%; Fig. 2) without heterogeneity or publication bias across the included studies (Fig. 6 ). After the meta-analysis model was adjusted to a random model, there was still a difference between the stem cell-based therapy group and the control group (RR = 0.75, 95% CI = 0.63–0.89, Additional Figure S1).

**Fig. 3:**
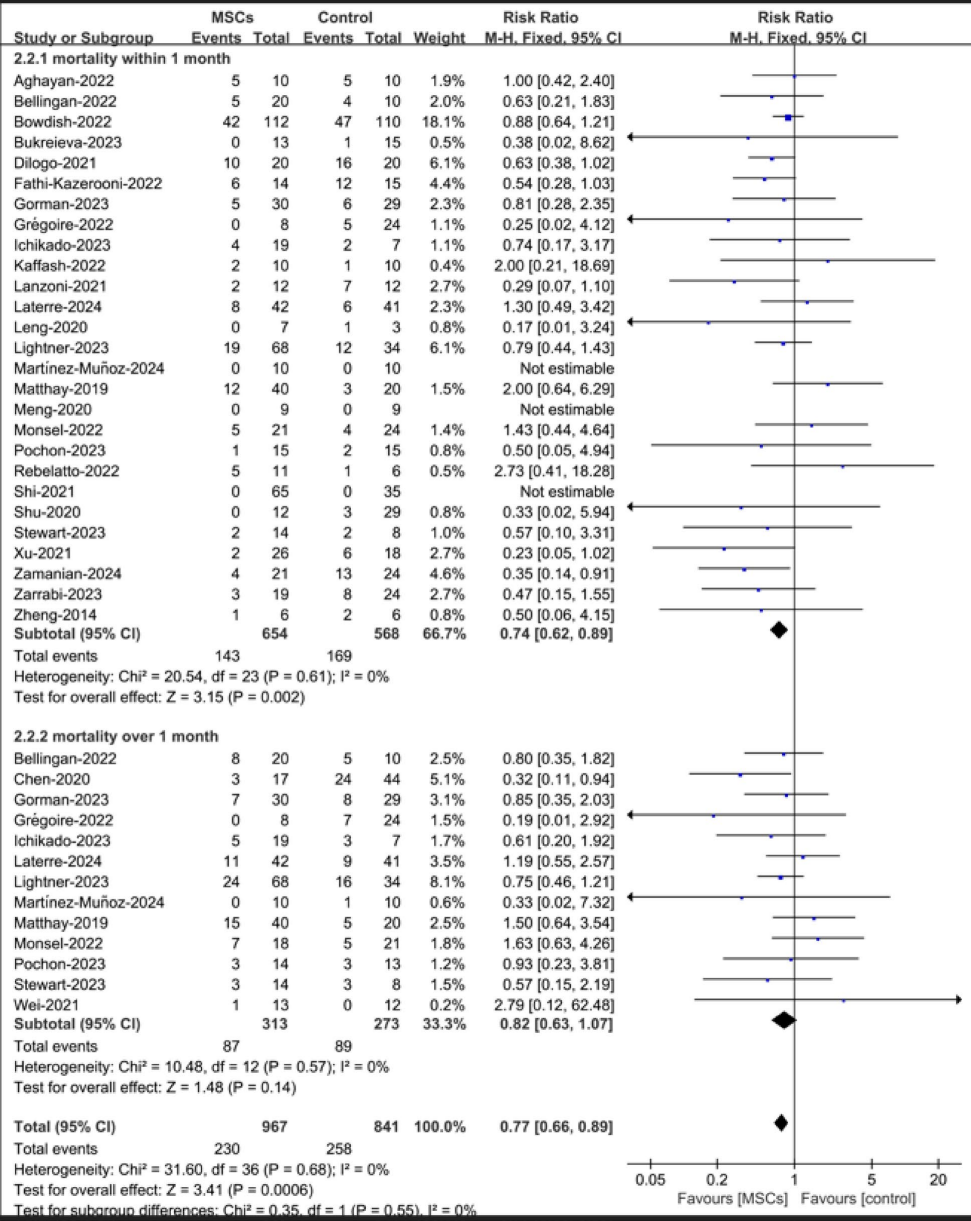
Forest plot of the subgroup of all-cause mortality according to the follow-up times

Next, mortality was assessed over different time periods. There were 27 studies reporting all-cause mortality within 1 month; 143 out of 654 patients in the stem cell-based therapy group died within 1 month, and 169 out of 568 patients in the control group died within 1 month. The difference between these groups was significant (RR = 0.74, 95% CI = 0.62–0.89, *p* = 0.002, *I²*=0). Meanwhile, all-cause mortality over 1 month was reported in 10 studies; the number of deaths was 87 out of 313 patients in the stem cell-based therapy group and 89 out of 273 patients in the control group. The difference between these groups was not significant (RR = 0.82, 95% CI = 0.63–1.07, *p* = 0.14, *I²*=0; Fig. 3). Two studies reported on mortality at Day 7 and Day 14, and one study reported on mortality at Day 10. Compared with the standard therapy group, there was no reduction in the stem cell-based therapy group in all-cause mortality within 10 days (RR = 1.15, 95% CI = 0.46–2.83, *p* = 0.77, *I²*=13%) or 14 days (RR = 0.94, 95% CI = 0.57–1.55, *p* = 0.82, *I²*=0; Additional Figure S1).

**Fig. 4:**
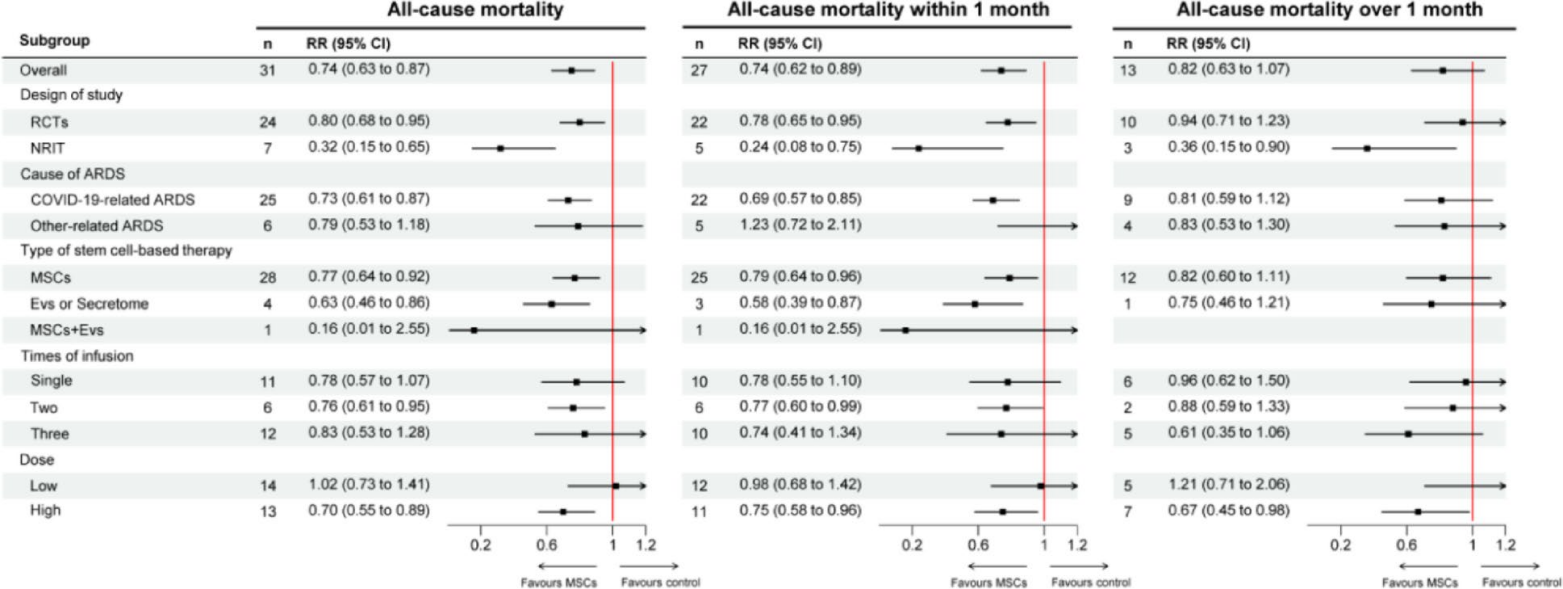
Subgroup analysis of all-cause mortality. RR: relative risk, RCT: randomized controlled trial, NRIT: non-randomized interventional trial, ARDS: acute respiratory distress syndrome, MSCs: mesenchymal stem cells, EVs: extracellular vesicles

In addition, when the studies were stratified according to the type of stem cell-based therapy, we found that MSCs reduced all-cause mortality of ARDS (RR = 0.77, 95% CI = 0.64–0.92, *p* = 0.005, *I*^*2*^ = 3%). Similar effects were observed for all-cause mortality within one month (RR = 0.79, 95% CI = 0.64–0.96, *p* = 0.02, *I²*=0) and over one month (RR = 0.82, 95% CI = 0.60–1.11, *p* = 0.20, *I²*=0). Besides, we also found EVs and secretomes reduced the risk of all-cause mortality within 1 month by 42% in 3 studies involving 176 ARDS patients (RR = 0.58, 95% CI = 0.39–0.87, *p* = 0.009, *I²*=7%; Fig. 4).

Regarding the cause and categories of ARDS, meta-analysis of 25 of the included studies shows that stem cell-based therapy was associated with a reduction in all-cause mortality for COVID-19-related ARDS (COVID-19-related ARDS: RR = 0.73, 95% CI = 0.61–0.87, *p* = 0.0005, *I²*=7%; other-related ARDS: RR = 0.79, 95% CI = 0.53–1.18, *p* = 0.26, *I*^*2*^ = 23%), especially within one month (RR = 0.69, 95% CI = 0.57–0.85, *p* = 0.0003, *I²*=0) (Fig. 4, additional figure S2).

Lastly, we attempt to explore how the times and dose of MSCs affected outcomes of ARDS. Although no statistical differences were found, multiple MSC infusions may reduce all-cause mortality with one month (one: RR = 0.78, 95% CI = 0.55–1.10; two: RR = 0.77, 95% CI = 0.60–0.99; and three: RR = 0.74, 95% CI = 0.41–1.34). Furthermore, high dose MSCs (over 1 × 10^6^ cells/kg or 7 × 10^7^ cells per infusion) was associated with reduction of all-cause mortality in ARDS (RR = 0.70, 95% CI = 0.55–0.89), and this protective effect remained significant when analyzed mortality within one month (RR = 0.75, 95% CI = 0.58–0.96).

### Meta-analysis of adverse events

**Fig. 5:**
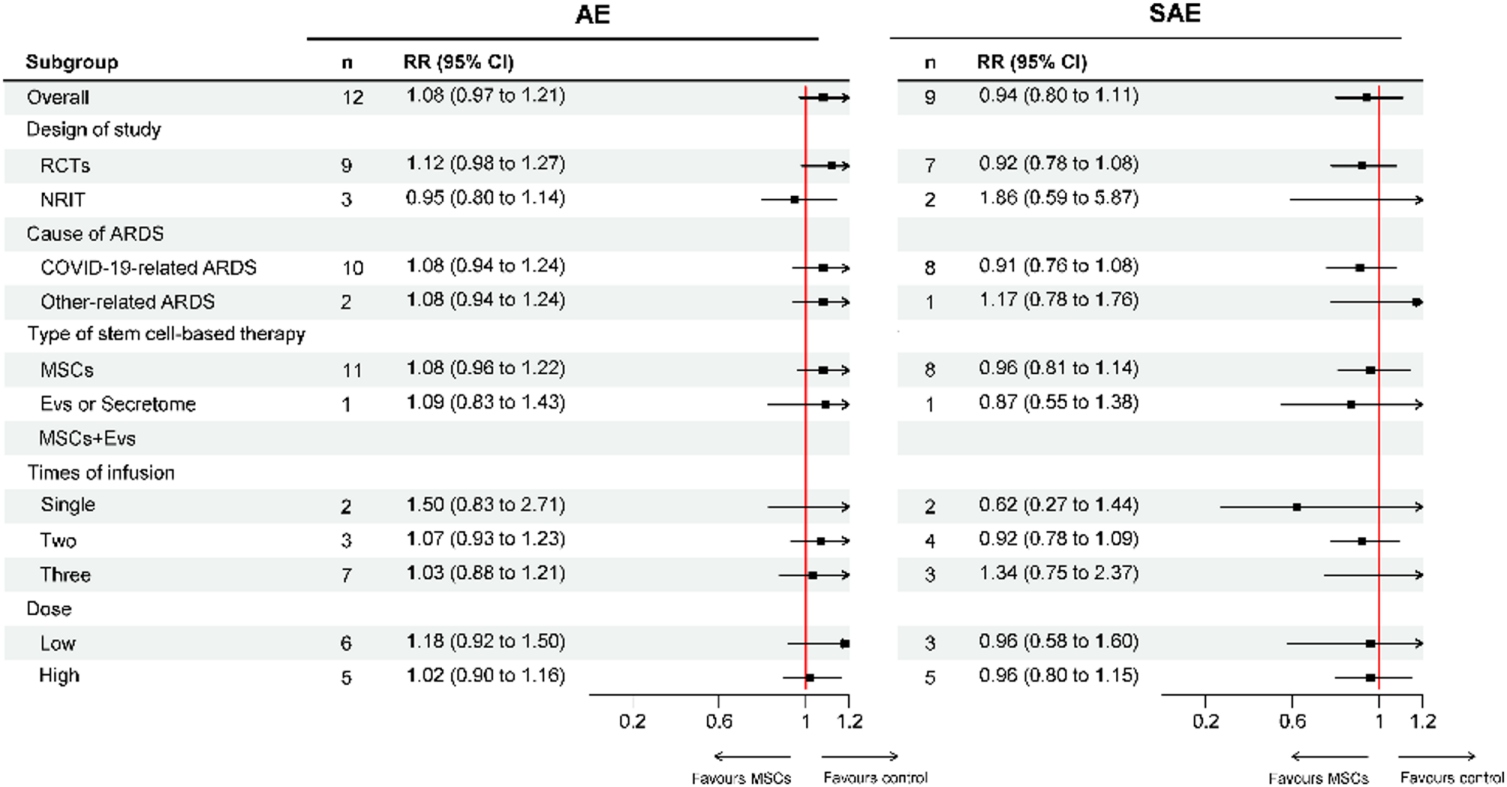
Subgroup analysis of AEs and SAEs. AE: adverse event, SAE: serious adverse event, RR: relative risk: RCT: randomized controlled trial, NRIT: non-randomized interventional trial, ARDS: acute respiratory distress syndrome, MSCs: mesenchymal stem cells, EVs: extracellular vesicles

**Fig. 6:**
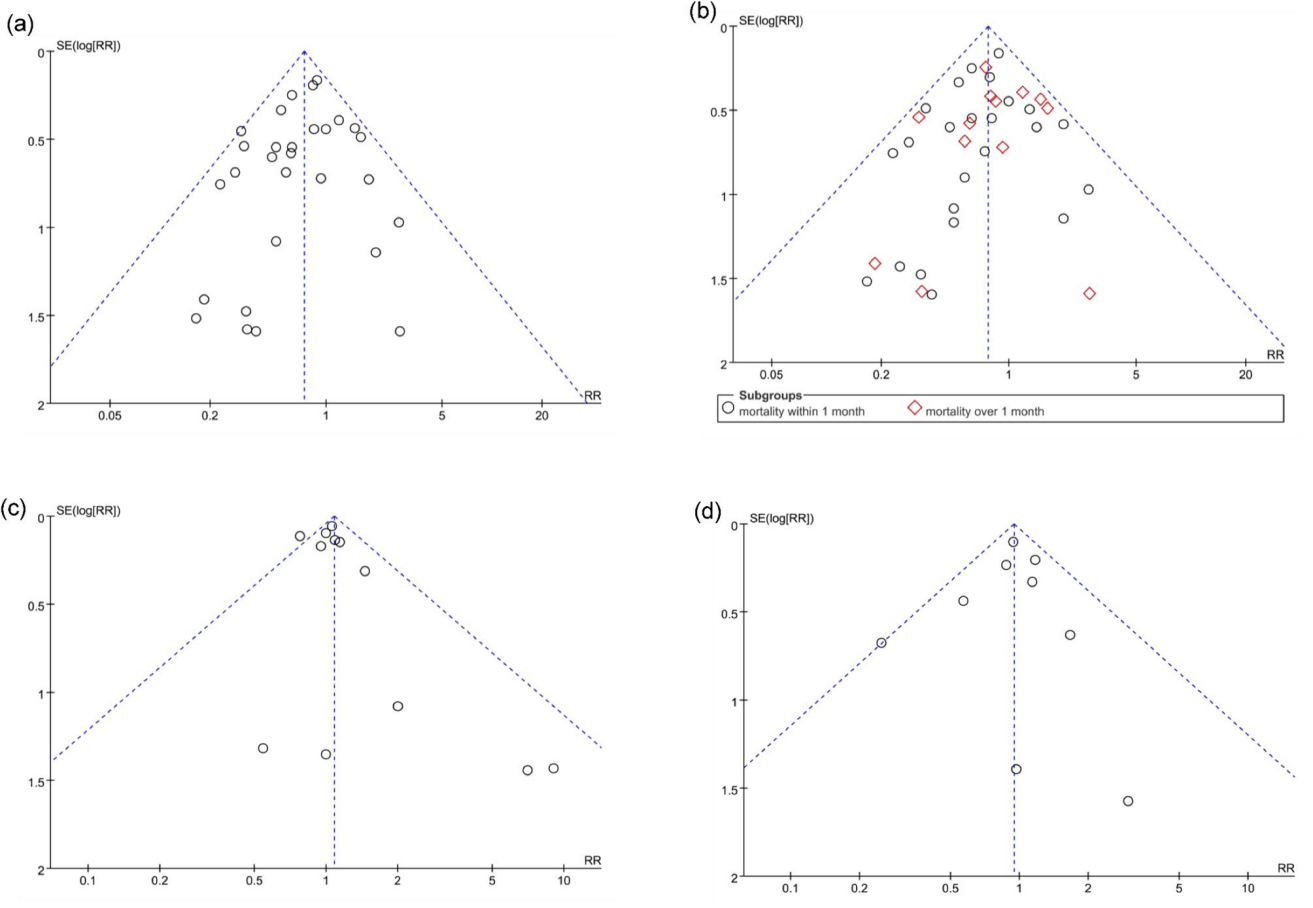
Publication bias assessment of (a) overall all-cause mortality, (b) the subgroup of all-cause mortality according to follow-up times, (c) AEs, and (d) SAEs. AE: adverse event, SAE: serious adverse event, RR: relative risk, SE: standard error

AE was measured in 12 studies with a total of 471 patients. The most common AE reported by included studies mainly included fever and shivering. Headache was also reported in a few studies, which was eliminated spontaneously or after complementary therapies. However, no difference was found between the stem cell-based therapy and control groups (RR = 1.08, 95% CI = 0.97–1.21, *p* = 0.17, *I*^*2*^ = 26%), indicating that stem cell-based therapy didn’t increase the incidence of AE. Besides, SAE was measured in 9 studies with a total of 495 patients. The pooled results showed that stem cell-based therapy did not increase the incidence of SAE (RR = 0.94, 95% CI = 0.80–1.11, *p* = 0.49, *I*^*2*^ = 0). As shown in Figs. 5 and 6 and a fixed-model was used for the AE and SAE analyses, and no heterogeneity was found.

In addition, subgroup analysis showed that MSCs (AE: RR = 1.08, 95% CI = 0.96–1.22; SAE: RR = 0.96, 95% CI = 0.81–1.14) and EVs (AE: RR = 1.09, 95% CI = 0.83–1.43; SAE: RR = 0.87, 95% CI = 0.55–1.38) didn’t increase the incidence of AE or SAE. Receiving high dose of MSCs (over 1 × 10^6^ cells/kg or 7 × 10^7^ cells per infusion) was not associated with higher AE or SAE incidence (AE: RR = 1.02, 95% CI = 0.90–1.16; SAE: RR = 0.96, 95% CI = 0.80–1.15). No difference on AE or SAE incidence was found between the stem cell-based therapy and control groups after one (AE: RR = 1.50, 95% CI = 0.83–2.71; SAE: RR = 0.62, 95% CI = 0.27–1.44), two (AE: RR = 1.07, 95% CI = 0.93–1.23; SAE: RR = 0.92, 95% CI = 0.78–1.09), and three (AE: RR = 1.03, 95% CI = 0.88–1.21; SAE: RR = 1.34, 95% CI = 0.75–2.37) infusions. (Fig. 5).

### Meta-analysis of additional outcomes

Considering the heterogeneity of ARDS, we also assessed additional efficacy outcomes besides all-cause mortality, including ventilator-free days, duration of ventilation, ICU-free days within one month, length of ICU stay, and days of hospitalization. five studies reported on the days of hospitalization, six studies reported on the length of ICU stay, four studies reported on the duration of ventilation, five reported on ICU-free days within one month, and nine studies reported on ventilator-free days within one month. As shown in Table 2 and additional Figures S3 and S4, no differences between MSC therapy and the standard therapy were detected for these variables (days of hospitalization: MD = 0.05, 95% CI=-6.26–6.36, *p* = 0.99, *I*^*2*^ = 73%; length of ICU stay: MD=-0.04, 95% CI=-2.78–2.71, *p* = 0.98, *I*^*2*^ = 16%; ventilation-free days within one month (MD=-0.46, 95% CI=-3.06–2.14, *p* = 0.73, *I*^*2*^ = 0; duration of ventilation for survivors (MD = 1.98, 95% CI=-5.37–9.33, *p* = 0.60, *I*^*2*^ = 74%; ICU-free days within one month (MD=-1.76, 95% CI=-5.55–2.03, *p* = 0.36, *I*^*2*^ = 0).

**Table 2:** Pooled results of the additional outcomes

Additional outcomes	*n*	participants	MD	95%CI	I^2^
Days of hospitalization	5	176	0.05	-6.26, 6.36	73%
Duration of ventilation	4	194	1.98	-5.37, 9.33	74%
ICU-free days within 1 month	5	156	-1.76	-5.55, 2.03	0%
Length of stay in the ICU	6	415	-0.04	-2.78, 2.71	16%
Ventilator-free days within 1 month	9	516	-0.46	-3.06, 2.14	0

n: number of studies included; MD: mean difference

Additional evidence for safety and efficacy of MSCs and its-derived EVs.

Overall, there were 14 studies on MSCs and 3 studies on EVs exploring the safety and efficacy of therapy. 13 studies found the positive effect of MSCs therapy on PaO_2_/FiO_2_ ratio or Oxygen saturation [58–63, 66–70, 72]. 7 studies reported the improvement of chest imaging after infusion or nebulization [62, 64, 66]^69^– ^71^, ^73^]. In 8 studies, the association was noted between the reduction of IL-6 or CRP and MSCs therapy [58–61, 65– 67, 69, 70]. Besides, three studies on EVs also reported CRP reduction after EVs treatment [71–73]. In terms of safety, 16 studies reported on safety profile and found no SAEs during or after MSC infusion. Only 4 studies found mild and transient adverse events (fever, pyrexia, transient liver enzyme elevation, headache) but resolved without sequelae [62, 63, 66, 70]. Additional evidence from single-arm, case report and case series studies were shown in Table 3.

**Table 3:** Additional evidence for safety and efficacy of MSCs and their -derived EVs

Study	Routine	Chest imaging	Laboratory effects	Clinical treatment outcome
Simonson-2015 [58]	intravenous infusion	-	↓Proinflammatory cytokines, miRNAs, and chemokines, epithelial apoptosis; Improved respiratory function with resolution of alveolar-capillary fluid leakage.	Safety: No AEs during MSC infusion. Efficacy: Both patients improved, with improved pulmonary compliance.
Chen-2022 [59]	intravenous infusion	-	↓CRP and MDW, IL-6, IL-12p70, IL-13, and VEGF; ↑PaO_2_/FiO_2_ ratio, total bilirubin; temporarily increased but normalized.	Safety: No AEs related to MSC infusion were observed. Efficacy: 3 discharged, 1 died from complications.
Haberle-2021 [60]	intravenous infusion	-	↓CRP and IL-6, leukocytes and neutrophils, Murray score; ↑Lymphocyte count, PaO_2_/FiO_2_ ratio.	Safety: No serious infusion-related AEs were reported. Efficacy: 80% survival rate compared to 45% in the control group, improved oxygenation and inflammation markers.
Brown-2022 [61]	intravenous infusion	-	↓CRP; ↑PaO_2_/FiO_2_ ratio, SOFA score.	-
Wilson-2015 [62]	intravenous infusion	-	↓Plasma IL-6, IL-8, and Ang-2, LIS, SOFA score; ↑PaO₂/FiO₂ ratio.	Safety: No pre-specified infusion-associated AEs, treatment-related SAEs occurred. Efficacy: 28-day mortality was lower than the expected rate for moderate ARDS.
Yip-2020 [63]	intravenous infusion	Number of lobar consolidations reduced on chest imaging,	↓Circulating inflammatory biomarkers, SOFA; ↑CD4⁺ T cells, CD8⁺ T cells, CD4⁺CD25⁺FOXp3⁺ regulatory T cells, PaO₂/FiO₂ ratio.	Safety: Transient AEs occurred in some patients but resolved without sequelae, no serious infusion-related adverse events were reported. Efficacy: In-hospital mortality was lower than the typical mortality with conventional therapy for severe ARDS.
Gorman-2021 [64]	intravenous infusion	-	No significant trends in IL-6, IL-8, IL-18, or SP-D; ↑PaO_2_/FiO_2_ ratio.	Safety: No dose-limiting toxicity; mild AEs (pyrexia, transient liver enzyme elevation) possibly related to the infusion, no serious adverse events reported at 1-year follow-up. Efficacy: 44% day-28 mortality.
Feng-2021 [65]	intravenous infusion	Chest imaging showed reduced infiltrates	↑lymphocytes, CD4, CD8, NK.	Safety: No acute infusion-related reactions, allergic responses, or delayed adverse events. Efficacy: Improved oxygenation, reduced inflammation, Mortality was lower than historical data.
Tao-2020 [66]	intravenous infusion	-	↓CRP, serum creatinine, blood urea nitrogen; ↑Lymphocyte count, pulmonary static compliance.	Safety: No febrile, allergic, or hemolytic reactions were observed during or after infusions. Efficacy: Delayed disease deterioration; showed limited but positive effects on respiratory and renal function.
Hashemian-2021 [67]	intravenous infusion	Lung CT showed reduced opacities.	↓ TNF-α, IL-8, and CRP; ↑SpO₂.	Safety: No SAEs; mild shivering in 2 cases. Efficacy: 55% survival rate with rapid improvement in respiratory distress.
Guo-2020 [68]	intravenous infusion	-	↓CRP, procalcitonin, IL-6, and D-dimer; ↑ Lymphocyte count, PaO_2_/FiO_2_ ratio.	Safety: No MSC-related AEs. Efficacy: 96.8% viral clearance; 87.1% discharged.
Ercelen-2021 [69]	intravenous infusion	-	↑SaO_2_	Safety: No severe MSC-related AEs. Efficacy: 61% overall survival rate: 52.5% of intubated patients and 77.5% of unintubated patients were discharged, patients treated with UC-MSCs before intubation had higher survival rates.
Zhang-2020 [70]	intravenous infusion	Chest CT showed reduced ground-glass opacities and infiltrates.	↓IL-6, TNF-α, CRP; ↑CD3⁺, CD4⁺, and CD8⁺ T cells, oxygen saturation.	Safety: No acute infusion-related reactions or delayed AEs were observed. Efficacy: Rapid symptoms (fever, dyspnea) resolved, patient was discharged 7 days post-infusion.
Saleh-2021 [71]	intravenous infusions	Chest CT showed reduced lung involvement.	↓IL-6, TNF-α, VEGF, and TGF-β, Ferritin; ↑IL-10, SDF-1, CD4⁺ and CD8⁺ T cells, oxygen saturation.	Safety: No serious complications; only a transient headache in 1 patient. Efficacy: 100% survival within 28 days.
Zhu-2022 [72]	nebulization	CT score reduced.	↓CRP, IL-6, LDH; ↑Lymphocyte counts.	Safety: No AEs or instability during/after nebulization. Efficacy: 4/7 patients showed obvious CT lesion resolution.
Sengupta − 2020 [73]	intravenous infusion	-	↓CRP, ferritin, and D-dimer, absolute neutrophil count; ↑Absolute lymphocyte count, PaO_2_/FiO_2_ ratio.	Safety: No AEs within 72 h; later events unrelated to EVs. Efficacy: 83% survival.
Chu-2022 [74]	nebulization	Pulmonary lesion absorption accelerated; mild cases had shorter absorption time than controls.	↑IFN-γ, IL-17 A and TH19; ↓CRP; ALT.	Safety: No acute or secondary allergic reactions; no adverse events. Efficacy: Promoted pulmonary lesion absorption, shortened hospitalization in mild cases.

CRP: C-reactive protein; IL-6: Interleukin-6; SOFA: Sequential Organ Failure Assessment; ALT: Alanine aminotransferase; MDW: monocyte distribution width; SAEs: serious adverse events; AEs: adverse events; LIS: Lung Injury Score; SDF-1: stromal cell-derived factor; SaO_2_: Arterial oxygen saturation; SpO₂: pulse Oxygen Saturation; SP-D: Surfactant protein-D; VEGF: vascular endothelial growth factor; -: no information

## Discussion

This comprehensive meta-analysis systematically evaluated stem cell-based therapy for ARDS treatment, revealing a significant reduction in mortality within the first month without increasing AEs or SAEs. Notably, EVs and secretomes showed preliminary efficacy, indicating their potential as therapeutic strategies for ARDS management.

Preclinical models and phase I trials have suggested safety and potential benefit of MSCs on ARDS [61, 74, 75]. Consist with clinical studies [16, 39, 42, 44, 52, 53], the reduction in all-cause mortality was observed within the first month of treatment. However, in the study with the largest sample size so far (222 patients included) [43], no statistically significant difference on the mortality rate within 30 days between these groups was found, with 37.5% in the stem cell-based therapy group and 42.7% in the control group reported. This may be attributed to the fact that many patients had progressed to a stage of inflammatory parenchymal lung damage, which was likely less amenable to modification through immunomodulation alone. The inconsistent findings of the selected studies regarding stem cell-based therapy for ARDS illustrate the need for a comprehensive examination of the underlying factors contributing to therapeutic response variability. First, the selected studies had small sample sizes and were not designed to have enough statistical power to evaluate clinical outcomes. Second, factors such as the MSC dose, source, and preparation and the timing of the infusion might contribute to variations in therapeutic efficacy [76, 77]. The present study explored of the impact of infusion times and dose on mortality in ARDS cases, suggesting the improved efficacy with over 1 × 10^6^ cells/kg or 7 × 10^7^ cells infusion, providing valuable insights for generating hypotheses for future phase III trial designs. Lastly, the variability in responses to stem cell-based therapy may be attributed to the inherent heterogeneity of ARDS, including baseline characteristics, such as the presence of hyperinflammatory or hypoinflammatory subphenotypes, as well as coexisting illnesses and concomitant medications [78–80]. The interaction between corticosteroids and MSCs remains uncertain [81, 82]. Li et al. [81]. found that continuous corticosteroid exposure may induce apoptosis in MSCs. But MSCs maintained immunomodulatory activity in combination with corticosteroids in graft-versus-host disease. However, although the uncertain interaction between corticosteroids and MSCs, the results so far proved that MSCs was safety in combination with corticosteroids. Given these complexities, future research should focus on optimizing treatment protocols and identifying the patient populations that are most likely to benefit from stem cell therapies. This includes standardizing stem cell preparation methods, discovering predictive biomarkers for treatment response, and conducting larger and more rigorous clinical trials that incorporate advanced statistical methodologies.

Given the severity of disease and the stem cells’ primary localization in the lung, AE assessment is paramount in ARDS patients received stem cell therapy. Previous studies have shown that a single high dose of MSCs (up to 10 × 10^6^ MSCs/kg) is well tolerated in COVID-19-related ARDS and other disease-related ARDS patients [14, 18, 48, 62]. However, a high dose has been associated with transient procoagulant effect [83] and may lead to a longer duration of mechanical ventilation [18]. In contrast, the repeated administration of low doses has been shown to have good tolerability, particularly among patients with COVID-19-related ARDS [40, 43]. So far, most AE reported were mild and could resolved spontaneously or after complementary therapies. This meta-analysis demonstrated a well-tolerated safety profile of MSCs infusion in ARDS patients. And further subgroup analysis revealed no increase AE in patients treated with over 1 × 10^6^ cells/kg or 7 × 10^7^ MSCs or repeated infusions. Notably, consist with findings with previous studies [20, 21, 33, 56], our results indicated that EVs and secretomes didn’t increase AE incidence as well.

Regarding on mechanism of MSCs therapy in lung injury, several clinical trials have yielded preliminary findings on the possible mechanism. The STem cells for ARDS Treatment (START) trial showed that MSC treatment significantly reduced the concentrations of angiopoietin-2 (Ang-2) in the airspace compared to the placebo. This reduction was independently associated with an increase in the number of days alive and a decrease in the need for mechanical ventilation [84]. In addition, the REALIST-COVID trial conducted a transcriptomic analysis of peripheral blood samples and differentially expressed genes (DEGs) indicated that MSCs modulate pathways related to cellular senescence, including the upregulation of unfolded protein responses and P53 signaling and the downregulation of sirtuin signaling [18, 80]. These findings collectively suggest that MSCs may play a multifaceted role in mitigating lung injury and promoting recovery in COVID-19 patients, warranting further investigation into their therapeutic potential and underlying mechanisms.

In addition, EVs have emerged as promising candidates for the treatment of ARDS due to their low immunogenicity, prolonged in vivo stability, high delivery efficiency, and minimal risk of inducing iatrogenic tumor formation [85, 86]. Four studies have demonstrated the preliminary safety and efficacy of EVs or secretome therapy in ARDS cases, and one study is currently recruiting patients (NCT05354141) [20, 21, 33, 56]. Zarrabi et al. [56] found that no patients died after receiving a combination of MSCs and EVs, while 3 of the 11 patients who only received MSCs died. And additional tests of inflammatory markers suggested that the combination of MSCs and EVs could serve as a suitable and accessible approach for alleviating the inflammatory cascade in COVID-19 patients. Furthermore, Lightner et al. [20] explored the effect of different doses of BM-MSCs-derived EVs (10 ml and 15 ml) in patients with moderate to severe ARDS. Their findings showed that the risk of 60-day mortality was significantly reduced in participants aged 18–65 years who received two doses of 15 ml of EVs compared to those who received the placebo. However, further research on EVs-related topics, such as the production of large-scale and standardized EVs and the optimal administration route and doses, is necessary before advancing to clinical studies to ensure reliable therapeutic outcomes in ARDS cases [85, 87, 88].

The present study had several limitations. First, as previously mentioned, all 31 clinical trials included are still in the phase 1/2 stage with limited sample size, making it difficult to conduct subgroup analysis. Although the effect of stem-cell therapy on overall all-cause mortality was detected, the statistical significance of the subgroup analysis results based on MSCs infusion times was not found, which may largely stemmed from the selected studies’ relatively small sample sizes. Second, despite our efforts to explore the effects of different frequencies of stem cell-based therapy, the variability in the MSC therapy schedules (including dose and timing of infusion) complicated the determination of the optimal therapeutic doses and infusion timing. This issue highlights the urgent need for trials comparing different stem cell therapy regimens for ARDS. Third, 80% (25/31) of the included studies focused on patients with COVID-19-related ARDS, it is noted to avoid extending their conclusions to non-COVID-19-related ARDS. Therefore, the findings of this meta-analysis are primarily applicable to COVID-19 patients. Forth, though high quality, studies so far on EVs were still limited with small sample size. Thus, the efficacy of EVs is preliminary and requires further validation.

## Conclusion

In summary, this meta-analysis demonstrated the safety and efficacy (reduction mortality within one month) of stem cell-based therapies for ARDS, although the heterogeneity of included patients, and provided valuable insights for designing future phase Ⅲ trials. Future research should focus on optimizing treatment protocols and investigating the underlying mechanisms to identify patients who are most likely to benefit from these innovative therapies.

## Supplementary Information

Below is the link to the electronic supplementary material.

Supplementary Material 1

Supplementary Material 2

Supplementary Material 3

Supplementary Material 4

Supplementary Material 5

Supplementary Material 6

Supplementary Material 7

Supplementary Material 8

## Data Availability

The datasets used and analyzed during the current study are included in this published article and its supplementary information files. Any query should be submitted to the corresponding author.

## Abbreviations

ARDS: Acute respiratory distress syndrome
AE: Adverse event
CI: Confidence interval
EVs: Extracellular vesicles
ICU: Intensive care units
MSCs: Mmesenchymal stem cells
MD: Mean difference
NRIT: Non-randomized interventional trial
RCT: Randomized controlled trial
RR: Relative risk
SAE: Serious adverse event
SD: Standard deviation
