# Supplementary material for: Efficacy and safety of mesenchymal stem/stromal cells and their derived extracellular vesicles for acute respiratory distress syndrome: a systematic review and meta-analysis
Source: Stem Cell Res Ther. 2025 Sep 29;16:522. doi: 10.1186/s13287-025-04644-4 (PMC12481956; doi:10.1186/s13287-025-04644-4)
Supplement: Supplementary file 1 — Supplementary Material 1 [file 13287_2025_4644_MOESM1_ESM.docx]

**Table S2: Search Strategy**

**PubMed**

**#1** "lung injury"[MeSH Terms] OR "Pneumonia"[MeSH Terms] OR "Respiratory Distress Syndrome"[MeSH Terms] OR "SARS-CoV-2"[MeSH Terms] OR "COVID-19"[MeSH Terms] OR "lung injury"[Title/Abstract] OR "lung damage"[Title/Abstract] OR "ALI"[Title/Abstract] OR "Pneumonia"[Title/Abstract] OR "Respiratory Distress Syndrome"[Title/Abstract] OR "ARDS"[Title/Abstract] OR "SARS-CoV-2"[Title/Abstract] OR "COVID-19"[Title/Abstract]

**#2**  "Mesenchymal Stem Cells"[MeSH Terms] OR "Stem Cells"[MeSH Terms] OR "Stromal Cells"[MeSH Terms] OR "Induced Pluripotent Stem Cells"[MeSH Terms] OR "mesenchymal stem cell"[Title/Abstract] OR "adult progenitor cell"[Title/Abstract] OR "stem cell"[Title/Abstract] OR "stromal cell"[Title/Abstract] OR "progenitor cell"[Title/Abstract] OR "msc"[Title/Abstract] OR "Induced Pluripotent Stem Cells"[Title/Abstract] OR "extracellular vesicle"[Title/Abstract] OR "iPS"[Title/Abstract] OR "iPSC"[Title/Abstract] OR "EVs"[Title/Abstract]

**#3**  "treatment outcome"[MeSH Terms] OR "death"[MeSH Terms] OR "mortality"[MeSH Terms] OR "survival"[MeSH Terms] OR "toxicity tests"[MeSH Terms] OR "drug tolerance"[MeSH Terms] OR "drug related side effects and adverse reactions"[MeSH Terms] OR "treatment outcome"[Title/Abstract] OR "death"[Title/Abstract] OR "mortality"[Title/Abstract] OR "survival"[Title/Abstract] OR "efficacy"[Title/Abstract] OR "effective*"[Title/Abstract] OR "effect"[Title/Abstract] OR "Treatment"[Title/Abstract] OR "Safety"[Title/Abstract] OR "adverse event"[Title/Abstract] OR "adverse drug event"[Title/Abstract] OR "adverse reaction"[Title/Abstract] OR "safe*"[Title/Abstract] OR "side effect"[Title/Abstract] OR "tolerance"[Title/Abstract] OR "toxicity"[Title/Abstract] OR "AE"[Title/Abstract] OR "SAE"[Title/Abstract] OR "death"[Title/Abstract] OR "die"[Title/Abstract]

**#4** "Patients"[Title/Abstract] OR "subjects"[Title/Abstract] OR "adults"[Title/Abstract] OR "cases"[Title/Abstract]

**#5** #1 AND #2 AND #3 AND #4

**Search Strategy for ClinicalTrials.Gov**

“Respiratory Distress Syndrome” OR “ARDS” OR “Coronavirus” OR "COVID 19" OR “pneumonia” OR “SARS-CoV-2” OR “lung injury” | “Extracelullar vesicle*” OR “Induced pluripotent cell*” OR iPS OR iPSC OR “stem cell*” OR “progenitor cell*” OR “Stromal Cells” OR MSC OR MSCs OR EVs | Adult (18 - 64), Older adult (65+)

**Search Strategy for Web of Science**

# Searches:

**1:** TS=("lung injury" OR "lung damage" OR "ALI" OR "Pneumonia" OR "Respiratory Distress Syndrome" OR "ARDS" OR "SARS-CoV-2" OR "COVID-19")

**2:** TS=("mesenchymal stem cell*" OR "adult progenitor cell*" OR "stem cell*" OR "stromal cell*" OR "progenitor cell*" OR "MSCs" OR "Induced Pluripotent Stem Cells" OR "extracelluar vesicle" OR "iPS" OR "iPSC" OR "EVs")

**3:** TS=("death" OR "Mortality" OR "Survival" OR "Treatment outcome $" OR “die $” OR "mortality" OR "survival" OR "efficacy" OR "effective *" OR "effect" OR "adverse event $" OR "adverse drug event $" OR "adverse reaction" OR "safe *" OR "AE $" OR "SAE $" OR "side effect" OR "tolerance" OR "toxicity")

**4**: (DT=(Editorial Material)) OR DT=(Review)

**5:** (((TS=(patients)) OR TS=(subjects)) OR TS=(adults)) OR TS=(cases)

**6:** #1 AND #2 AND #3 AND #5

**7:** #6 NOT #4

**Search Strategy for Cochrane Library**

**#1** MeSH descriptor: [Mesenchymal Stem Cells] explode all trees

**#2** MeSH descriptor: [Stromal Cells] explode all trees

**#3** MeSH descriptor: [Stem Cells] explode all trees

**#4** MeSH descriptor: [Extracellular Vesicles] explode all trees

**#5** (mesenchymal stem cell OR adult progenitor cell OR stem cell OR stromal cell OR progenitor cell OR MSCs OR extracellular vesicle OR iPS OR iPSC OR EVs):ti,ab,kw (Word variations have been searched)

**#6** MeSH descriptor: [Lung Injury] explode all trees

**#7** MeSH descriptor: [Pneumonia] explode all trees

**#8** MeSH descriptor: [Respiratory Distress Syndrome] explode all trees

**#9** MeSH descriptor: [SARS-CoV-2] explode all trees

**#10** MeSH descriptor: [COVID-19] explode all trees

**#11** ("lung injury" OR "lung damage" OR "ALI" OR "Pneumonia" OR "Respiratory Distress Syndrome" OR "ARDS" OR "SARS-CoV-2" OR "COVID-19"):ti,ab,kw (Word variations have been searched)

**#12** #6 OR #7 OR #8 OR #9 OR #10 OR #11

**#13** #1 OR #2 OR #3 OR #4 OR #5

**#14** #12 AND #13
