# Supplementary material for: Efficacy and safety of mesenchymal stem/stromal cells and their derived extracellular vesicles for acute respiratory distress syndrome: a systematic review and meta-analysis
Source: Stem Cell Res Ther. 2025 Sep 29;16:522. doi: 10.1186/s13287-025-04644-4 (PMC12481956; doi:10.1186/s13287-025-04644-4)
Supplement: Supplementary file 3 — Supplementary Material 3 [file 13287_2025_4644_MOESM3_ESM.docx]

**Table S3. Quality assessment of randomized studies according to the Cochrane Risk of Bias tool 1.0**

| **Study** | **Selection bias** | | **Performance bias** | **Detection bias** | **Attrition bias** | **Reporting bias** | **Other bias** |
| --- | --- | --- | --- | --- | --- | --- | --- |
|  | Random sequence generation | Allocation concealment | Blinding of participants and personnel | Blinding of outcome assessment | Incomplete outcome data | Selective reporting | Other sources of bias |
| Adas-2021 | unclear | high | low | low | low | low | low |
| Aghayan-2022 | high | high | high | high | low | low | Low |
| Bellingan-2022 | unclear | low | low | low | low | low | low |
| Bowdish-2022 | low | low | low | low | low | low | low |
| de Dios-2023 | low | high | low | low | high | high | high |
| Dilogo-2021 | low | low | low | low | low | low | low |
| Fathi-Kazerooni-2022 | low | unclear | low | low | low | high | high |
| Gorman-2023 | low | low | low | low | low | low | low |
| Ichikado-2023 | unclear | low | high | high | high | low | low |
| Kaffash-2022 | unclear | unclear | high | high | low | low | unclear |
| Lanzoni-2021 | low | low | low | low | low | low | low |
| Laterre-2024 | low | low | low | low | high | low | high |
| Lighter-2023 | unclear | low | low | low | low | low | low |
| Martínez-Muñoz-2024 | low | low | low | low | low | low | unclear |
| Matthay-2019 | low | low | low | low | low | low | low |
| Monsel-2022 | unclear | low | low | low | low | low | low |
| Pochon-2023 | low | low | low | unclear | low | low | unclear |
| Rebelatto-2022 | unclear | low | low | low | unclear | low | unclear |
| Shi-2021 | low | low | low | low | low | low | unclear |
| Shu-2020 | unclear | unclear | high | high | low | low | low |
| Stewart-2023 | unclear | low | unclear | unclear | low | low | unclear |
| Zamanian-2024 | low | low | low | low | high | low | unclear |
| Zarrabi-2023 | low | low | high | high | low | low | unclear |
| Zheng-2014 | unclear | low | low | low | low | low | unclear |

Low risk, high risk, unclear risk

**Table S4. Quality assessment of non-randomized studies according to the ROBINS-I V2 tool.**

| **Study** | **Pre-intervention** | | **At intervention** | **Post-intervention** | | | |  |
| --- | --- | --- | --- | --- | --- | --- | --- | --- |
|  | **Bias due to confounding** | **Bias in the selection of participants into the study** | **Bias in the classification of interventions** | **Bias due to deviations from intended interventions** | **Bias due to missing data** | **Bias in measurement of the outcomes** | **Bias in selection of the reported result** | **Overall** |
| Leng-2020 | Moderate risk | Low risk | Low risk | Low risk | Low risk | Low risk | Moderate risk | Moderate risk |
| Meng-2020 | Moderate risk | Low risk | Low risk | Low risk | Low risk | Low risk | Low risk | Moderate risk |
| Xu-2021 | Moderate risk | Low risk | Low risk | Low risk | Low risk | Low risk | Low risk | Moderate risk |
| Wei-2021 | Low risk | Low risk | Low risk | Low risk | Low risk | Low risk | Moderate risk | Low risk |
| Chen-2020 | Low risk | Low risk | Low risk | Low risk | Low risk | Low risk | Low risk | Low risk |
| Bukreieva-2023 | Moderate risk | Low risk | Low risk | Low risk | Low risk | Low risk | Moderate risk | Moderate risk |
| Grégoire-2022 | Low risk | Low risk | Low risk | Low risk | Low risk | Low risk | Low risk | Low risk |
| Ercelen-2021 | Serious risk | Low risk | Low risk | Moderate risk | Low risk | Low risk | Low risk | Moderate risk |
| Feng-2021 | NI | Low risk | Low risk | Moderate risk | Low risk | Low risk | Low risk | Moderate risk |
| Gorman-2021 | Low risk | Low risk | Low risk | Moderate risk | Low risk | Low risk | Low risk | Moderate risk |
| Yip-2020 | NI | Low risk | Low risk | Moderate risk | Low risk | Low risk | Moderate risk | Moderate risk |
| Wilson-2015 | Low risk | Low risk | Low risk | Moderate risk | Low risk | Low risk | Low risk | Moderate risk |
| Haberle-2021 | Serious risk | Low risk | Low risk | Moderate risk | Low risk | Low risk | Low risk | Moderate risk |
| Chen-2022 | NI | Low risk | Low risk | Moderate risk | Low risk | Low risk | Low risk | Moderate risk |
| Saleh-2021 | NI | Low risk | Low risk | Moderate risk | Low risk | Low risk | Moderate risk | Moderate risk |
| Zhu-2022 | NI | Low risk | Low risk | Moderate risk | Low risk | Low risk | Low risk | Moderate risk |
| Sengupta -2020 | Serious risk | Low risk | Low risk | Moderate risk | Low risk | Low risk | Moderate risk | Moderate risk |
| Chu-2022 | NI | Low risk | Low risk | Moderate risk | Low risk | Low risk | Moderate risk | Moderate risk |

NI: no information.

**Table S5. Quality assessment of case report according to the Joanna Briggs institute (JBI).**

| **Study** | **Item 1** | **Item 2** | **Item 3** | **Item 4** | **Item 5** | **Item 6** | **Item 7** | **Item 8** | **Total score** |
| --- | --- | --- | --- | --- | --- | --- | --- | --- | --- |
| Simonson-2015 | Yes | Yes | Yes | Yes | Yes | Yes | Yes | Yes | 8 |
| Tao-2020 | Yes | Yes | Yes | Yes | Yes | Yes | Yes | Yes | 8 |
| Zhang-2020 | Yes | Yes | Yes | Yes | Yes | Yes | Yes | Yes | 8 |

NA, Not applicable

**Table S6. Quality assessment of case series according to the Joanna Briggs institute (JBI).**

| **Study** | **Item 1** | **Item 2** | **Item 3** | **Item 4** | **Item 5** | **Item 6** | **Item 7** | **Item 8** | **Item 9** | **Item 10** | **Total score** |
| --- | --- | --- | --- | --- | --- | --- | --- | --- | --- | --- | --- |
| Brown-2022 | Yes | Yes | Yes | Yes | No | Yes | No | Yes | No | Yes | 7 |
| Hashemian-2021 | Yes | Yes | Yes | No | Yes | Yes | Yes | Yes | Yes | Yes | 9 |
| Guo-2020 | Yes | unclear | Yes | Yes | No | Yes | Yes | Yes | No | Yes | 7 |

NA, Not applicable
