# Supplementary material for: Efficacy and safety of mesenchymal stem/stromal cells and their derived extracellular vesicles for acute respiratory distress syndrome: a systematic review and meta-analysis
Source: Stem Cell Res Ther. 2025 Sep 29;16:522. doi: 10.1186/s13287-025-04644-4 (PMC12481956; doi:10.1186/s13287-025-04644-4)
Supplement: Supplementary file 4 — Supplementary Material 4 [file 13287_2025_4644_MOESM4_ESM.docx]

**Table S7.** The coexisting illness, baseline cytokine levels and concomitant medication of included patients

| **Study**  **(Journal)** | **Coexisting illness**  (stem cell-based therapy group; control group), n (%) | **Baseline cytokine levels**  ( stem cell-based therapy group; control group), Mean ± SD/ median, (IQR) | **Concomitant Medication for ARDS**  (stem cell-based therapy group; control group), n (%) |
| --- | --- | --- | --- |
| Zheng-2014(1)  (*Respir Res*) | Hypertension: 3(50); 3(50)  Coronary artery disease: 1(16.67); 1(16.67)  Neurologic disease: 5(83.330; 3(50)  Chronic pulmonary disease: 1(16.67); 0  Diabetes: 2(33.33); 1(16.67) | - | - |
| Matthay-2019(2)  (*Lancet Respir Med*) | - | IL-6: 167 (61, 541); 164 (30, 300) pg/mL  CRP (pg/mL): 60 (44, 76); 75 (58, 105) mg/L | - |
| Lanzoni-2021(3)  (*Stem Cells Transl Med*) | Diabetes: 5(41.7);6(50)  Hypertension: 7(58.3);9(75)  Obesity (BMI >30): 11(91.7); 5(41.7)  Cancer: 0; 1 (8.3)  Heart disease: 1(8.3); 3(25) | - | Heparin: 12(100); 12(100)  Remdesivir: 9(75); 7(58.3)  Convalescent plasma: 3 (25); 4(33.3)  Corticosteroids: 10(83.3); 9(75)  Tocilizumab:1(8.3); 4(33.3)  Hydroxychloroquine: 1(8.3); 2(18.2)  Alteplase: 0; 2(16.7) |
| Dilogo-2021(4)  (*Stem Cells Transl Med*) | Diabetes:8(40); 12(60)  Hypertension: 6(30); 10(50)  Chronic kidney disease: 2(10); 5(25)  Coronary arterial disease: 2(10); 3(15)  Congestive heart failure:1(5); 1(5)  Tuberculosis: 1(5); 1(5)  Others^a^: 10(50); 6(30) | - | - |
| Shi-2021(5)  (*Signal Transduct Target Ther*) | Hypertension: 17(26.15); 10(28.57)  Diabetes: 27(18.46); 12(14.29)  Chronic bronchitis: 2(3.08); 3(8.57)  COPD: 2(3.08); 0 | IL-6: 7.86 (5.63, 9.84); 8.76 (6.54, 11.77) pg/mL  CRP: 1.95 (0.84, 3.53) 1.38 (0.68, 2.26) mg/L | Antiviral drugs: 32(49.23); 20(57.14)  Antibiotics: 27(41.54); 12(34.29)  Corticosteroids: 13(20.00); 9(25.71) |
| Adas-2021(6)  (*Cell Transplant*) | - | - | - |
| Monsel-2022(7)  (*Crit Care*) | COPD: 0; 1(6.7)  Active smoking: 0; 0  Chronic heart failure: 0; 0  Atrial fibrillation: 2(13.3); 0  Hypertension: 11(73.3); 10(66.7)  Coronary artery disease: 2(13.3); 2(13.3)  Stroke: 2(13.3); 1(6.7)  Immunodeficiency: 0; 0  Active neoplasia: 0; 0 | - | Corticosteroid: 0; 0  Immunomodulatory drugs: 2(11.8); 0 |
| Rebelatto-2022(8)  (*Stem Cell Res Ther*) | Diabetes: 4(36.4); 3(50)  Hypertension: 6(54.5); 3(50)  Kidney disease: 1(9.1); 0  COPD: 0; 1(16.7)  Schizophrenia: 1(9.1); 0  Obesity (BMI > 30): 6(54.5); 3(50) | - | Anticoagulant: 11(100); 6(100)  Steroids: 11(100); 6(100)  Antibiotics: 2(18.2); 1(16.7)  Antiviral drugs: 2(18.2); 0 |
| Aghayan-2022(9)  (*Stem Cell Res Ther*) | Diabetes: 3(30); 4(40)  Hypertension: 4(40); 4(40)  Interstitial Lung Disease: 1(10); 0  Irritable Bowel Syndrome: 1(10); 0  Chronic kidney disease: 0; 1(10)  Sarcoidosis: 1(10); 10  Asthma: 1(10); 1(10)  Rheumatoid Arthritis: 1(10); 0  Liver hemangioma: 0; 1(10) | - | - |
| Shu-2020(10)  (*Stem Cell Res Ther*) | Diabetes: 3 (25); 5 (17.24)  Hypertension: 3(33.33); 6(20.69) | - | Antibiotic agent: 10(83.33): 26(89.66)  Antiviral treatment: 12(100); 29(100)  Vasopressors: 0; 7(24.14)  Glucocorticoid: 12(100); 29(100) |
| Bowdish-2022(11)  (*Am J Respir Crit Care Med*) | Hypertension: 65(59.1); 63(58.9)  Diabetes: 46(42.2); 42(39.3)  Renal disease: 14(12.8); 13(12.1)  Prior pulmonary disease: 18(16.5); 15(14.2)  Heart failure: 6(5.5); 4(3.7)  Cancer: 12(11.0); 11(10.4)  History of smoking/e-cigarette/Vaping: 35(34.7); 31(30.7) | CRP: 130 (66, 215); 152 (88, 223) mg/L  IL-6: 58.2 (23.2, 248.9); 56.0 (22.1, 205.2) pg/mL | Remdesivir: 76(67.9); 74(67.3)  Convalescent plasma: 25(22.3); 30(27.3)  IL-6 inhibitor: 6(5.4); 5(4.5)  Glucocorticoid: 92(82.1); 96(87.3)  Antibiotics: 92(82.1); 83(75.5)  Anticoagulation: 103(92.0); 106(96.4)  Antiplatelets: 22(19.6); 20(18.2)  ACE inhibitor or ARB: 4(3.6); 1(0.9)  Neuromuscular blockade or paralytic: 75(67.0); 71(64.5)  Pulmonary vasodilators: 16(14.3); 15(13.6) |
| Kaffash-2022(12)  (*Stem Cell Res Ther*) | Hypertension:3(30); 1(10)  Diabetes: 1(10); 2(20)  Anemia: 0; 1(10) | CRP: 107.73 ± 7.2; 83.36 ± 9.4 mg/L  IL‑6: 121.65 ± 25.78; 138.906 ± 30.68 pg/mL | - |
| Leng-2020(13)  (*Aging Dis*) | - | - | - |
| Meng-2020(14)  (*Signal Transduct Target Ther*) | Hypertension: 2(22.22); 1(11.11)  Diabetes: 1(11.11); 0  Fatty liver disease: 1(11.11); 0  Asthma: 0; 1(11.11) | - | LPV/r: 8(88.89); 8(88.89),  glucocorticoid: 9(100); 6(66.67) |
| Xu-2021(15)  (*Clin Transl Med*) | - | - | Symptomatic treatment: 24(92.31); 18(100)  Antiviral therapy: 23(88.46); 18(100)  Antibacterial treatment: 15(57.70); 18(100)  Hormone: 14(53.85); 14(77.78)  Gut microflora modulator: 1(3.85); 4(22.22)  Extracorporeal blood purification system: 10(38.46); 9(50)  Traditional Chinese medicine: 15(57.70); 13(72.22)  Basic disease medication: 5(19.23); 7(38.89) |
| Wei-2021(16)  (*Clinics*) | Diabetes: 1(8.33)  Hemorrhagic cerebral infarction: 1(8.33) | - | Arbidol: 12(100),  LPV/r: 12(100),  methylprednisolone: 4(33.33) |
| Chen-2020(17)  (*Engineering*) | Hypertension: 10 (58.8); 23 (52.3)  Diabetes: 5 (29.4); 7 (15.9)  Coronary heart diseases: 0; 8(18.2)  COPD: 0; 1(2.3)  Chronic kidney disease: 0; 2(4.5)  Hematological diseases: 0; 1(2.3)  Cancer: 0; 4(9.1)  Liver diseases: 1 (5.9); 1 (2.3)  Renal failure: 1 (9); 10 (22.7)  Shock: 12 (70.6); 16 (36.4)  Intestinal diseases: 5 (29.4); 5 (11.4)  Double pneumonia: 17 (100); 41 (93.2) | CRP: 98.96 ± 97.03; 124.56 ± 89.64 mg/L | Antiviral agent: 17 (100); 44 (100)  Antibiotic therapy: 14 (82.4); 36 (81.8)  Vasoactive drugs: 12 (70.6); 19 (43.2)  Glucocorticoid: 9 (52.9); 24 (54.5) |
| Bukreieva-2023(18)  (*Int J Mol Sci*) | Hypertension: 5(38.5); 9(60.0)  Diabetes: 1(7.7); 1(6.67)  Heart disease: 4(30.7); 6(40.0) | - | - |
| Zarrabi-2023(19)  (*Stem Cell Res Ther*) | Having comorbidities: 5 (45.5); 3 (37.5); 11 (45.83) | IL-6: 209.4 ± 56.4; 207.2 ± 38.9; 147.5 ± 56.2 pg/mL  CRP: 36.9 ± 15.36; 25.3 ± 10.2; 30.2 ± 14.3 mg/L | Dexamethasone: 8 (72.7); 6 (75); 19 (79.2)  Prednisolone: 3 (27.3); 2 (25); 5 (20.8)  Enoxaparin: 7 (63.6); 5 (62.5); 17 (70.8)  Heparin: 4 (36.4); 3 (37.5); 7 (29.2)  Remdesivir: 2 (18.2); 1 (12.5); 5 (20.8) |
| Ichikado-2023(20)  (*Stem Cell Res Ther*) | - | - | - |
| Bellingan-2022(21)  (*Intensive Care Med.*) | - | - | Corticosteroids:12(60); 8(80）  Vasopressor: 9(45); 3(30) |
| Gorman-2023(22)  (*Am J Respir Crit Care Med*) | - | - | Corticosteroid: 27(90); 26(89.7)  IL-6 blockade: 0; 0  Lopinavir/Ritonavir: 2(6.7); 3(10.3)  Remedesivir: 11(36.7); 48(48.30)  LPV/r:2 (6.7); 3 (10.3) |
| Pochon-2023(23)  (*Front Med* ) | Chronic heart failure: 0; 1(7)  Hypertension: 5(33);10(67)  Diabetes: 3(20);4(27) | - | Corticosteroids: 6 (40);9 (60)  Vasopressors: 6 (40);7 (47) |
| Grégoire-2022(24)  (*Front Immunol*) | - | CRP: 171.6 (127.5-212.2); 126.5 (62.2-216.5) mg/L | Dexamethasone: 8(100); 24(100)  Anticoagulation: 8(100); 24(100) |
| Zamanian-2024(25)  (*J Extracell Vesicles*) | - | - | - |
| Lightner-2023(26)  (*chest*) | - | - | Remdesivir: 17 (50.0); 21(61.8);23 (67.6)  Plasma: 7 (20.6); 9 (26.5); 9 (26.5)  Dexamethasone: 26 (76.5); 25 (73.5); 27 (79.4) |
| Stewart-2023  (abstract) | - | - | - |
| Martínez-Muñoz-2024(27)  (*Bone Marrow Transplant*) | Diabetes: 4(40); 2(20)  Obesity: 3(30); 6(60)  Cardiovascular disorder (including HBP): 9(90); 6(60)  Chronic lung disease: 1(10); 2(20) | IL-6: 307.95 ± 380.18; 450.22 ± 555.46 pg/mL | Remdesivir:0; 1(10)  Glucocorticoid: 10(100); 10(100)  Tocilizumab: 10(100);9(90)  Low molecular weight heparin: 10(100); 10(100) |
| Laterre-2024(28)  (*J Crit Care*) | - | - | β-lactam antibacterials: 12 (28.6); 21 (51.2)  Macrolides, lincosamides, and Streptogramins: 19 (45.2); 21 (51.2) Quinolone: 19 (45.2); 15 (36.6)  Corticosteroids: 19 (45.2); 12 (29.3) |
| Fathi-Kazerooni-2022(29)  (*Stem Cell Res Ther*) | Diabetes: 3(21.5);4(26.6)  Hypertension: 4(28.5); 5(33.3) | - | Remdesivir:15(100);14(100) Corticosteroid:15(100);14(100)  Anticoagulants:15(100);14(100) |
| de Dios-2023(30)  (*Front Med*) | - | - | - |
| Simonson-2015(31)  (*Stem Cells Transl Med*) | Hypertension: 1 (50)  Acute kidney failure: 1 (50)  Liver failure: 1 (50)  Acute myeloid leukemia: 1 (50)  Severe thrombocytopenia: 1 (50) | - | Oseltamivir: 1 (50)  Broad-spectrum antibiotics: 1 (50) |
| Chen-2022(32)  (*Front Med*) | Diabetes: 1 (25); 1 (25)  Hypertension: 1 (25); 1 (25)  Hyperlipidemia: 1 (25); 1 (25)  Coronary artery disease: 0; 1 (25)  Solid-organ malignancy: 0; 1 (25) | CRP: 7.25 (3.35–13.16); 6.32 (0.63–11.73) mg/dL | Remdesivir: 4 (100); 4 (100)  Dexamethasone: 4 (100); 4 (100)  Tocilizumab: 4 (100): 4 (100) |
| Haberle-2021(33)  (*J Intensive Care Med*) | hypertension: 1 (20); 13 (72) | CRP: 18.5 (16.6–35.3); 28.3 (21.6–34.5) mg/L  IL-6: 144 (82.6–899.6); 443.5 (77.4–845.3) pg/mL | - |
| Brown-2022(34)  (*Cytotherapy*) | Obesity: 8 (73)  Diabetes mellitus: 3 (27)  Hypertension: 3 (27)  Asthma: 2 (18) | - | Hydroxychloroquine: 11 (100)  Tocilizumab: 5 (45)  Glucocorticoids: 8 (73)  Vasopressors: 6 (55) |
| Wilson-2015(35)  (*Lancet Respir Med*) | - | IL-6: 762 (419–1,198) pg/mL  IL-8: 35 (18–48.5) pg/mL  Ang-2: 7,507 (3,977–14,950) pg/mL | - |
| Yip-2020(36)  (*Crit Care Med*) | Hypertension: 3(33.3)  Type 2 diabetes mellitus: 3 (33.3)  Chronic kidney disease: 5(55.6)  Chronic hepatitis/cirrhosis: 2 (22.2) | CRP: 166.4 ± 116.7 mg/L | - |
| Gorman-2021(37)  (*EClinicalMedicine*) | - | IL-6: 62.2 (21.1–84.6) pg/mL  IL-8: 61.3 (6.1–59.2) pg/mL  IL-18: 44.4 (16.1–133.7) pg/mL  Ang-2: 61.3 (6.1–59.2) pg/mL  ICAM-1: 54.3 (4.2–59.2) pg/mL  SP-D: 59.2 (5.5–59.2) ng/mL | - |
| Feng-2021(38)  (*Cell Prolif*) | Hypertension: 2 (22.2); 0  Diabetes mellitus: 2 (22.2); 1 (14.3)  Alzheimer's disease: 0; 1 (14.3) | - | - |
| Tao-2020(39)  (*J Infect Dev Ctries*) | Type 2 diabetes mellitus: 1 (100)  Diabetic nephropathy: 1 (100)  Renal insufficiency: 1 (100)  Hypertension: 1 (100) | CRP: 149.42 mg/L | Recombinant human interferon: 1 (100)  Lopinavir/ritonavir: 1 (100)  Methylprednisolone: 1 (100) |
| Hashemian-2021(40)  (*Stem Cell Res Ther*) | Hypertension: 3 (27.3)  Diabetes mellitus: 3 (27.3)  Cardiomyopathy: 1 (9.1)  Chronic lymphocytic leukemia: 1 (9.1) | - | Hydroxychloroquine: 9 (81.8)  lopinavir/ritonavir: 11 (100)  Azithromycin: 2 (18.2)  Meropenem: 4 (36.4)  Vancomycin: 4 (36.4)  Intravenous immunoglobulin: 2 (18.2)  Ribavirin: 2 (18.2)  Favipiravir: 2 (18.2) |
| Guo-2020(41)  (*Crit Care*) | Hypertension: 13 (41.9)  COPD: 6 (19.4)  Coronary artery disease: 5 (16.1)  Diabetes mellitus: 5 (16.1) | CRP: 13.39 (1.30–38.86) mg/L  IL-6: 13.78 (5.69–25.26) pg/mL | Antivirals: 26 (83.9)  Arbidol: 20 (64.5)  Interferon alfa-2b: 9 (29.0)  Oseltamivir: 3 (9.7)  Chloroquine: 3 (9.7)  Antibiotics: 23 (74.2)  Methylprednisolone: 6 (19.4) |
| Ercelen-2021(42)  (*Stem Cell Rev Rep*) | - | - | - |
| Zhang-2020(43)  (*Stem Cell Res Ther*) | Diabetes mellitus: 1 (100) | CRP: 59.64 mg/L | Lopinavir/ritonavir: 1 (100)  Inhaled IFN-α: 1 (100) |
| Saleh-2021(44)  (*Stem Cell Res Ther*) | Fatty liver disease: 1 (20)  Hypertension: 1 (20)  Damaged lung: 1 (20)  Diabetes mellitus: 1 (20) | - | Heparin: 5 (100)  Dexamethasone: 5 (100)  Atazanavir: 1 (20) |
| Zhu-2022(45)  (*Stem Cell Res Ther*) | Hypertension: 3 (42.9)  Diabetes mellitus: 2 (28.6)  COPD: 1 (14.3)  Hyperthyroidism: 1 (14.3) | CRP: 35 (18–52) mg/L  IL-6: 45 (25–70) pg/mL | Arbidol: 5 (71.4)  Interferon-α: 6 (85.7)  Cefoperazone/tazobactam: 5 (71.4)  Meropenem: 4 (57.1)  Methylprednisolone: 3 (42.9)  Convalescent plasma: 4 (57.1) |
| Sengupta -2020(46)  (*Stem Cells Dev*) | Pre-diabetes: 3 (12.5)  Type 2 diabetes mellitus: 20 (83.3)  Hypertension: 12 (50.0)  Hyperlipidemia: 5 (20.8) | CRP: 1007 ± 720 mg/L | Hydroxychloroquine: 24 (100)  Azithromycin: 24 (100) |
| Chu-2022(47)  (*Stem Cell Rev Rep*) | Liver damage: 1 (14.3)  Fatty liver: 1 (14.3)  Diabetes mellitus: 1 (14.3) | CRP: 23.00 ± 31.87 mg/L | corticosteroid: 2 (28.6) |

COPD: chronic obstructive pulmonary disease; CRP: C-reactive protein (mg/L); IL-6: Interleukin-6; Ang-2: angiopoietin-2; SP-D: Surfactant protein-D; LPV/r: lopinavir/ritonavir, ^a^Other comorbidities include gastric perforation, pleural effusion, multiple rib fractures, obesity, hypercoagulation, and lung contusion in the Recovered group and icterus, stroke infarction, Disseminated Intravascular Coagulation, atrial fibrillation, obesity, acute kidney injury, myocardial infarction, and hypertensive heart disease in the Died group. -: no information.

1. Zheng G, Huang L, Tong H, Shu Q, Hu Y, Ge M, et al. Treatment of acute respiratory distress syndrome with allogeneic adipose-derived mesenchymal stem cells: a randomized, placebo-controlled pilot study. Respir Res. 2014;15(1):39.

2. Matthay MA, Calfee CS, Zhuo H, Thompson BT, Wilson JG, Levitt JE, et al. Treatment with allogeneic mesenchymal stromal cells for moderate to severe acute respiratory distress syndrome (START study): a randomised phase 2a safety trial. The Lancet Respiratory medicine. 2019;7(2):154-62.

3. Lanzoni G, Linetsky E, Correa D, Messinger Cayetano S, Alvarez RA, Kouroupis D, et al. Umbilical cord mesenchymal stem cells for COVID-19 acute respiratory distress syndrome: A double-blind, phase 1/2a, randomized controlled trial. Stem cells translational medicine. 2021;10(5):660-73.

4. Dilogo IH, Aditianingsih D, Sugiarto A, Burhan E, Damayanti T, Sitompul PA, et al. Umbilical cord mesenchymal stromal cells as critical COVID-19 adjuvant therapy: A randomized controlled trial. Stem cells translational medicine. 2021;10(9):1279-87.

5. Shi L, Huang H, Lu X, Yan X, Jiang X, Xu R, et al. Effect of human umbilical cord-derived mesenchymal stem cells on lung damage in severe COVID-19 patients: a randomized, double-blind, placebo-controlled phase 2 trial. Signal transduction and targeted therapy. 2021;6(1):58.

6. Adas G, Cukurova Z, Yasar KK, Yilmaz R, Isiksacan N, Kasapoglu P, et al. The Systematic Effect of Mesenchymal Stem Cell Therapy in Critical COVID-19 Patients: A Prospective Double Controlled Trial. Cell transplantation. 2021;30:9636897211024942.

7. Monsel A, Hauw-Berlemont C, Mebarki M, Heming N, Mayaux J, Nguekap Tchoumba O, et al. Treatment of COVID-19-associated ARDS with mesenchymal stromal cells: a multicenter randomized double-blind trial. Critical care (London, England). 2022;26(1):48.

8. Rebelatto CLK, Senegaglia AC, Franck CL, Daga DR, Shigunov P, Stimamiglio MA, et al. Safety and long-term improvement of mesenchymal stromal cell infusion in critically COVID-19 patients: a randomized clinical trial. Stem cell research & therapy. 2022;13(1):122.

9. Aghayan HR, Salimian F, Abedini A, Ghazi SF, Yunesian M, Alavi-Moghadam S, et al. Human placenta-derived mesenchymal stem cells transplantation in patients with acute respiratory distress syndrome (ARDS) caused by COVID-19 (phase I clinical trial): safety profile assessment. Stem Cell Res Ther. 2022;13(1).

10. Shu L, Niu CM, Li RY, Huang TR, Wang Y, Huang M, et al. Treatment of severe COVID-19 with human umbilical cord mesenchymal stem cells. Stem Cell Res Ther. 2020;11(1).

11. Bowdish ME, Barkauskas CE, Overbey JR, Gottlieb RL, Osman K, Duggal A, et al. A Randomized Trial of Mesenchymal Stromal Cells for Moderate to Severe Acute Respiratory Distress Syndrome from COVID-19. Am J Respir Crit Care Med. 2023;207(3):261‐70.

12. Kaffash Farkhad N, Sedaghat A, Reihani H, Adhami Moghadam A, Bagheri Moghadam A, Khadem Ghaebi N, et al. Mesenchymal stromal cell therapy for COVID-19-induced ARDS patients: a successful phase 1, control-placebo group, clinical trial. Stem cell research & therapy. 2022;13(1):283.

13. Leng Z, Zhu R, Hou W, Feng Y, Yang Y, Han Q, et al. Transplantation of ACE2(-) Mesenchymal Stem Cells Improves the Outcome of Patients with COVID-19 Pneumonia. Aging and disease. 2020;11(2):216-28.

14. Meng FP, Xu RN, Wang SY, Xu Z, Zhang C, Li YY, et al. Human umbilical cord-derived mesenchymal stem cell therapy in patients with COVID-19: a phase 1 clinical trial. Signal transduction and targeted therapy. 2020;5(1).

15. Xu X, Jiang W, Chen L, Xu Z, Zhang Q, Zhu M, et al. Evaluation of the safety and efficacy of using human menstrual blood-derived mesenchymal stromal cells in treating severe and critically ill COVID-19 patients: An exploratory clinical trial. Clinical and translational medicine. 2021;11(2):e297.

16. Wei FT, Kong DX, Li T, Li A, Tan Y, Fang JF, et al. Efficacy and safety of umbilical cord mesenchymal stem cells for the treatment of patients with COVID-19. Clinics. 2021;76.

17. Chen J, Hu C, Chen L, Tang L, Zhu Y, Xu X, et al. Clinical Study of Mesenchymal Stem Cell Treatment for Acute Respiratory Distress Syndrome Induced by Epidemic Influenza A (H7N9) Infection: A Hint for COVID-19 Treatment. Engineering (Beijing, China). 2020;6(10):1153-61.

18. Bukreieva T, Svitina H, Nikulina V, Vega A, Chybisov O, Shablii I, et al. Treatment of Acute Respiratory Distress Syndrome Caused by COVID-19 with Human Umbilical Cord Mesenchymal Stem Cells. Int J Mol Sci. 2023;24(5).

19. Zarrabi M, Shahrbaf MA, Nouri M, Shekari F, Hosseini SE, Hashemian SR, et al. Allogenic mesenchymal stromal cells and their extracellular vesicles in COVID-19 induced ARDS: a randomized controlled trial. Stem cell research & therapy. 2023;14(1):169.

20. Ichikado K, Kotani T, Kondoh Y, Imanaka H, Johkoh T, Fujimoto K, et al. Clinical efficacy and safety of multipotent adult progenitor cells (invimestrocel) for acute respiratory distress syndrome (ARDS) caused by pneumonia: a randomized, open-label, standard therapy-controlled, phase 2 multicenter study (ONE-BRIDGE). Stem cell research & therapy. 2023;14(1):217.

21. Bellingan G, Jacono F, Bannard-Smith J, Brealey D, Meyer N, Thickett D, et al. Safety and efficacy of multipotent adult progenitor cells in acute respiratory distress syndrome (MUST-ARDS): a multicentre, randomised, double-blind, placebo-controlled phase 1/2 trial. Intensive Care Med. 2022;48(1):36‐44.

22. Gorman EA, Rynne J, Gardiner HJ, Rostron AJ, Bannard-Smith J, Bentley AM, et al. Repair of Acute Respiratory Distress Syndrome in COVID-19 by Stromal Cells (REALIST-COVID Trial): A Multicenter, Randomized, Controlled Clinical Trial. American journal of respiratory and critical care medicine. 2023;208(3):256-69.

23. Pochon C, Laroye C, Kimmoun A, Reppel L, Dhuyser A, Rousseau H, et al. Efficacy of Wharton Jelly Mesenchymal Stromal Cells infusions in moderate to severe SARS-Cov-2 related acute respiratory distress syndrome: a phase 2a double-blind randomized controlled trial. Frontiers in medicine. 2023;10:1224865.

24. Gregoire C, Layios N, Lambermont B, Lechanteur C, Briquet A, Bettonville V, et al. Bone Marrow-Derived Mesenchymal Stromal Cell Therapy in Severe COVID-19: Preliminary Results of a Phase I/II Clinical Trial. Front Immunol. 2022;13.

25. Zamanian MH, Norooznezhad AH, Hosseinkhani Z, Hassaninia D, Mansouri F, Vaziri S, et al. Human placental mesenchymal stromal cell-derived small extracellular vesicles as a treatment for severe COVID-19: A double-blind randomized controlled clinical trial. Journal of extracellular vesicles. 2024;13(7):e12492.

26. Lightner AL, Sengupta V, Qian S, Ransom JT, Suzuki S, Park DJ, et al. Bone Marrow Mesenchymal Stem Cell-Derived Extracellular Vesicle Infusion for the Treatment of Respiratory Failure From COVID-19: A Randomized, Placebo-Controlled Dosing Clinical Trial. Chest. 2023;164(6):1444-53.

27. Martínez-Muñoz ME, Payares-Herrera C, Lipperheide I, de Molina RM, Salcedo I, Alonso R, et al. Mesenchymal stromal cell therapy for COVID-19 acute respiratory distress syndrome: a double-blind randomised controlled trial. Bone Marrow Transplant. 2024;59(6):777-84.

28. Laterre PF, Sánchez García M, van der Poll T, Wittebole X, Martínez-Sagasti F, Hernandez G, et al. The safety and efficacy of stem cells for the treatment of severe community-acquired bacterial pneumonia: A randomized clinical trial. J Crit Care. 2024;79:154446.

29. Fathi-Kazerooni M, Fattah-Ghazi S, Darzi M, Makarem J, Nasiri R, Salahshour F, et al. Safety and efficacy study of allogeneic human menstrual blood stromal cells secretome to treat severe COVID-19 patients: clinical trial phase I & II. Stem cell research & therapy. 2022;13(1):96.

30. de Dios C, Vij R, Kim H, Park H, Chang D. Safety of multiple intravenous infusions of adipose-derived mesenchymal stem cells for hospitalized cases of COVID-19: a randomized controlled trial. Frontiers in medicine. 2023;10:1321303.

31. Simonson OE, Mougiakakos D, Heldring N, Bassi G, Johansson HJ, Dalén M, et al. In Vivo Effects of Mesenchymal Stromal Cells in Two Patients With Severe Acute Respiratory Distress Syndrome. Stem cells translational medicine. 2015;4(10):1199-213.

32. Chen CH, Chang KC, Lin YN, Ho MW, Cheng MY, Shih WH, et al. Mesenchymal stem cell therapy on top of triple therapy with remdesivir, dexamethasone, and tocilizumab improves PaO(2)/FiO(2) in severe COVID-19 pneumonia. Frontiers in medicine. 2022;9:1001979.

33. Häberle H, Magunia H, Lang P, Gloeckner H, Körner A, Koeppen M, et al. Mesenchymal Stem Cell Therapy for Severe COVID-19 ARDS. Journal of intensive care medicine. 2021;36(6):681-8.

34. Whittaker Brown SA, Iancu-Rubin C, Aboelela A, Abrahams A, Burke E, Drummond T, et al. Mesenchymal stromal cell therapy for acute respiratory distress syndrome due to coronavirus disease 2019. Cytotherapy. 2022;24(8):835-40.

35. Wilson JG, Liu KD, Zhuo H, Caballero L, McMillan M, Fang X, et al. Mesenchymal stem (stromal) cells for treatment of ARDS: a phase 1 clinical trial. The Lancet Respiratory medicine. 2015;3(1):24-32.

36. Yip HK, Fang WF, Li YC, Lee FY, Lee CH, Pei SN, et al. Human Umbilical Cord-Derived Mesenchymal Stem Cells for Acute Respiratory Distress Syndrome. Crit Care Med. 2020;48(5):e391-e9.

37. Gorman E, Shankar-Hari M, Hopkins P, Tunnicliffe WS, Perkins GD, Silversides J, et al. Repair of acute respiratory distress syndrome by stromal cell administration (REALIST) trial: A phase 1 trial. EClinicalMedicine. 2021;41:101167.

38. Feng Y, Huang J, Wu J, Xu Y, Chen B, Jiang L, et al. Safety and feasibility of umbilical cord mesenchymal stem cells in patients with COVID-19 pneumonia: A pilot study. Cell Prolif. 2020;53(12):e12947.

39. Tao J, Nie Y, Wu H, Cheng L, Qiu Y, Fu J, et al. Umbilical cord blood-derived mesenchymal stem cells in treating a critically ill COVID-19 patient. Journal of infection in developing countries. 2020;14(10):1138-45.

40. Hashemian SR, Aliannejad R, Zarrabi M, Soleimani M, Vosough M, Hosseini SE, et al. Mesenchymal stem cells derived from perinatal tissues for treatment of critically ill COVID-19-induced ARDS patients: a case series. Stem cell research & therapy. 2021;12(1):91.

41. Guo Z, Chen Y, Luo X, He X, Zhang Y, Wang J. Administration of umbilical cord mesenchymal stem cells in patients with severe COVID-19 pneumonia. Critical care (London, England). 2020;24(1):420.

42. N OE, Pekkoc-Uyanik KC, Alpaydin N, Gulay GR, Simsek M. Clinical experience on umbilical cord mesenchymal stem cell treatment in 210 severe and critical COVID-19 cases in Turkey. Stem cell reviews and reports. 2021;17(5):1917-25.

43. Zhang Y, Ding J, Ren S, Wang W, Yang Y, Li S, et al. Intravenous infusion of human umbilical cord Wharton's jelly-derived mesenchymal stem cells as a potential treatment for patients with COVID-19 pneumonia. Stem cell research & therapy. 2020;11(1):207.

44. Saleh M, Vaezi AA, Aliannejad R, Sohrabpour AA, Kiaei SZF, Shadnoush M, et al. Cell therapy in patients with COVID-19 using Wharton's jelly mesenchymal stem cells: a phase 1 clinical trial. Stem cell research & therapy. 2021;12(1):410.

45. Zhu YG, Shi MM, Monsel A, Dai CX, Dong X, Shen H, et al. Nebulized exosomes derived from allogenic adipose tissue mesenchymal stromal cells in patients with severe COVID-19: a pilot study. Stem cell research & therapy. 2022;13(1):220.

46. Sengupta V, Sengupta S, Lazo A, Woods P, Nolan A, Bremer N. Exosomes Derived from Bone Marrow Mesenchymal Stem Cells as Treatment for Severe COVID-19. Stem Cells Dev. 2020;29(12):747-54.

47. Chu M, Wang H, Bian L, Huang J, Wu D, Zhang R, et al. Nebulization Therapy with Umbilical Cord Mesenchymal Stem Cell-Derived Exosomes for COVID-19 Pneumonia. Stem cell reviews and reports. 2022;18(6):2152-63.
