# Supplementary material for: Efficacy and safety of mesenchymal stem/stromal cells and their derived extracellular vesicles for acute respiratory distress syndrome: a systematic review and meta-analysis
Source: Stem Cell Res Ther. 2025 Sep 29;16:522. doi: 10.1186/s13287-025-04644-4 (PMC12481956; doi:10.1186/s13287-025-04644-4)
Supplement: Supplementary file 5 — Supplementary Material 5 [file 13287_2025_4644_MOESM5_ESM.docx]

**Figure S1. Forest plot of all-cause mortality** **(calculated using random model) (A), all-cause mortality within 10 days (B), and all-cause mortality at day 14 (C)**

**
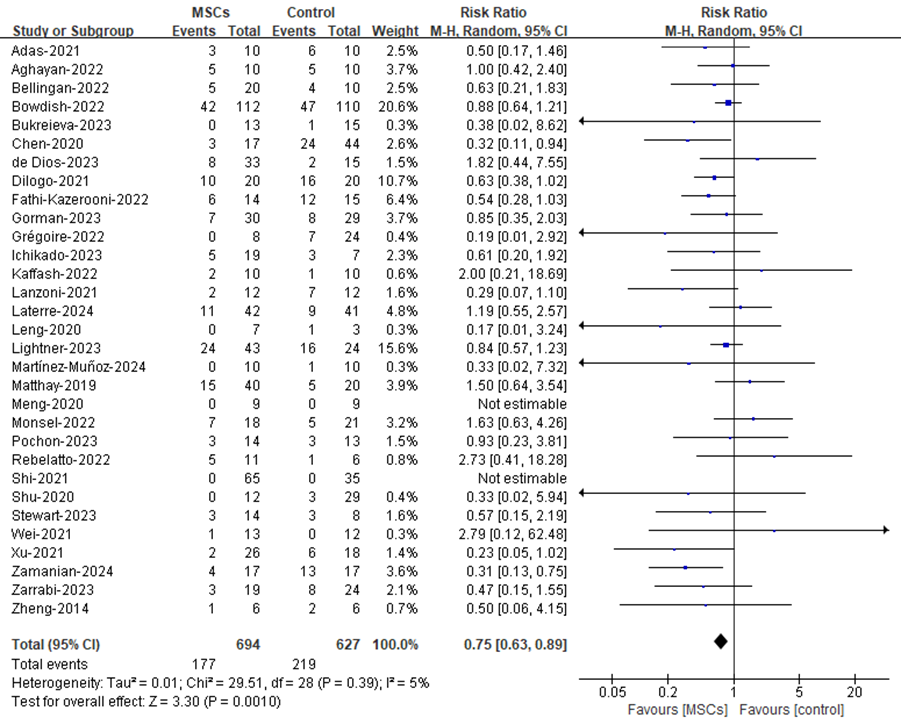
**

**A**


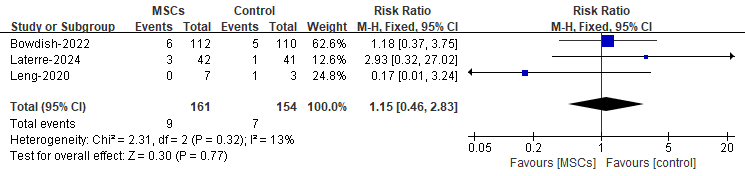


**B**


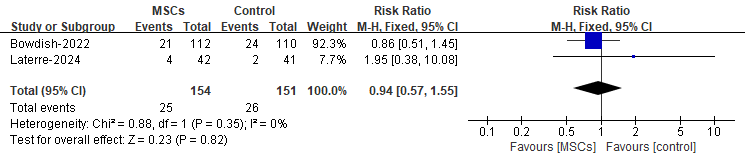


**C**
