# Supplementary material for: Efficacy and safety of mesenchymal stem/stromal cells and their derived extracellular vesicles for acute respiratory distress syndrome: a systematic review and meta-analysis
Source: Stem Cell Res Ther. 2025 Sep 29;16:522. doi: 10.1186/s13287-025-04644-4 (PMC12481956; doi:10.1186/s13287-025-04644-4)
Supplement: Supplementary file 6 — Supplementary Material 6 [file 13287_2025_4644_MOESM6_ESM.docx]

**Figure S2. Forest plot of subgroup analysis** **according to categories of ARDS of all-cause mortality (A), all-cause mortality within 1 month (B), all-cause mortality over 1 month (C), AEs (D), and SAEs (E)**


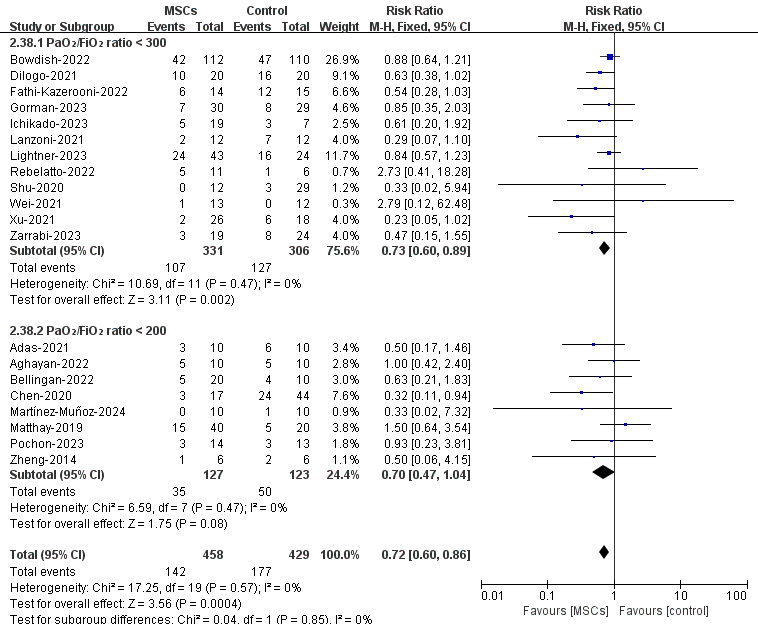


**A**


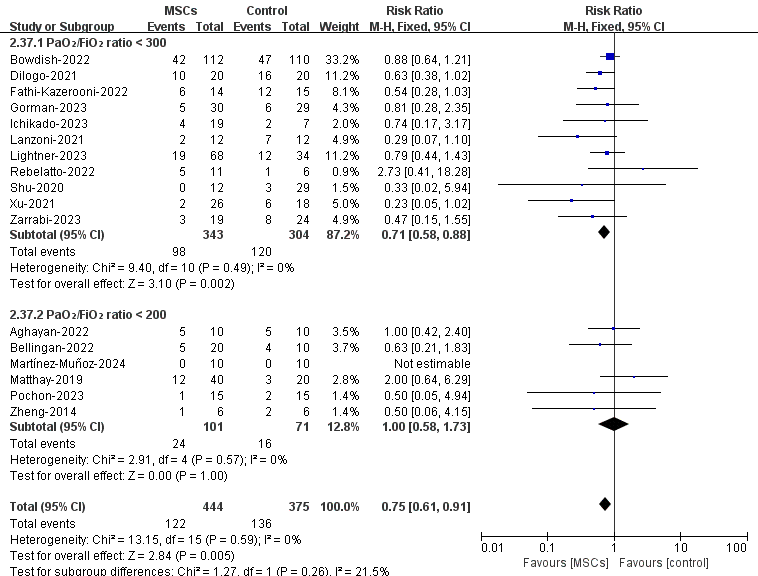


**B**


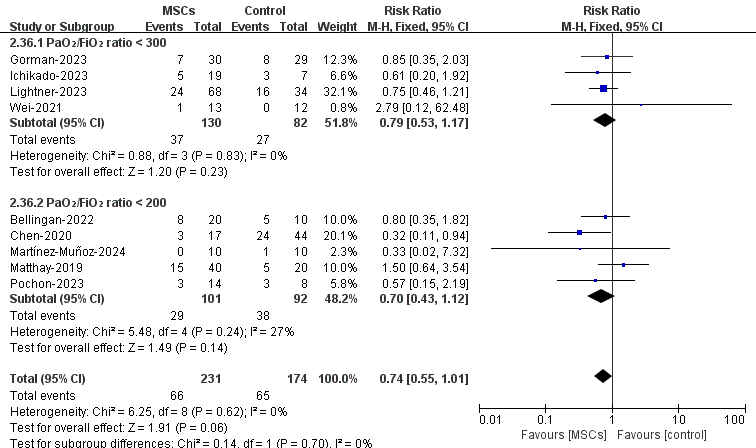


**C**


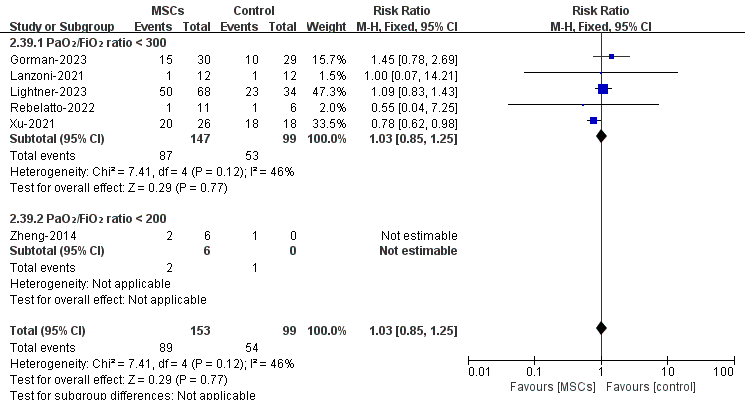


**D**


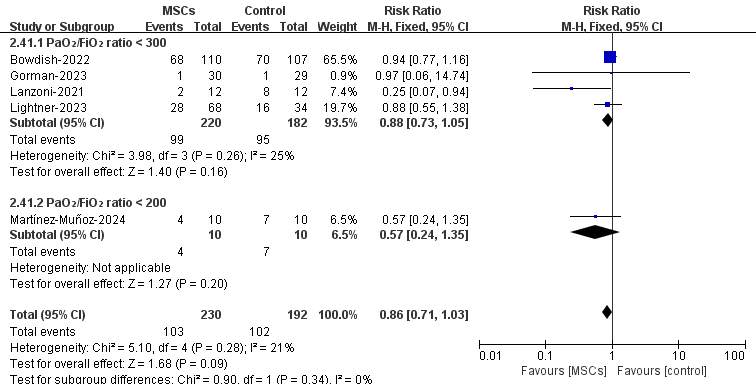


**E**
