# Supplementary material for: Efficacy and safety of mesenchymal stem/stromal cells and their derived extracellular vesicles for acute respiratory distress syndrome: a systematic review and meta-analysis
Source: Stem Cell Res Ther. 2025 Sep 29;16:522. doi: 10.1186/s13287-025-04644-4 (PMC12481956; doi:10.1186/s13287-025-04644-4)
Supplement: Supplementary file 7 — Supplementary Material 7 [file 13287_2025_4644_MOESM7_ESM.docx]

**Figure S3. Forest plot of days of hospitalization (A), duration of ventilation (B), ICU-free days within 1 month (C), length of stay in the ICU (D), and ventilation-free days within 1 month (E)**


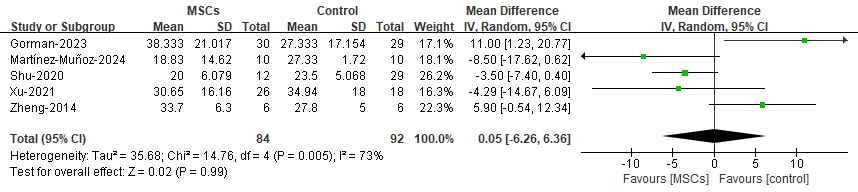
A


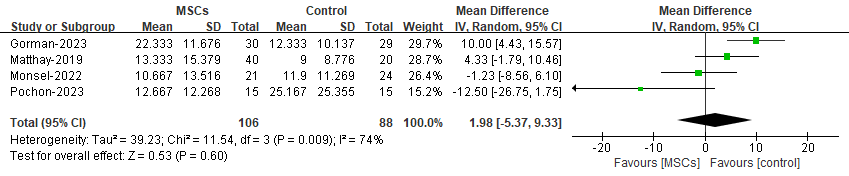
**B**


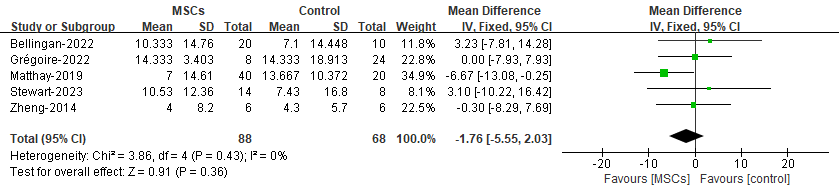
**C**


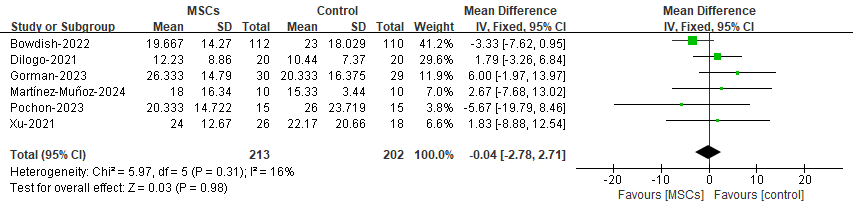
**D**


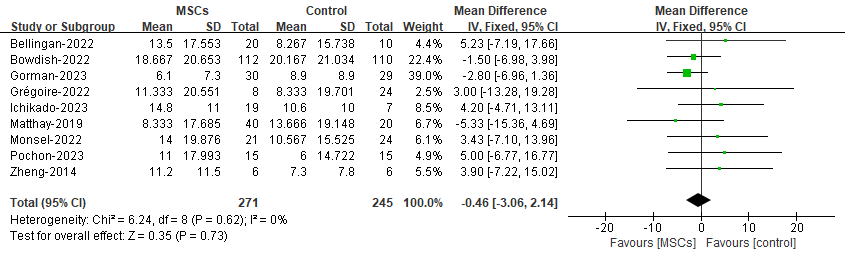
**E**
