# Supplementary material for: Efficacy and safety of mesenchymal stem/stromal cells and their derived extracellular vesicles for acute respiratory distress syndrome: a systematic review and meta-analysis
Source: Stem Cell Res Ther. 2025 Sep 29;16:522. doi: 10.1186/s13287-025-04644-4 (PMC12481956; doi:10.1186/s13287-025-04644-4)
Supplement: Supplementary file 8 — Supplementary Material 8 [file 13287_2025_4644_MOESM8_ESM.docx]

**Figure S4. Funnel plots of publication bias of** **ventilation-free days within 1 month (A), days of hospitalization (B), duration of ventilation in survivors (C), ICU-free days within 1 month (D), length of stay in the ICU (E), and ventilation-free days within 1month (F)**


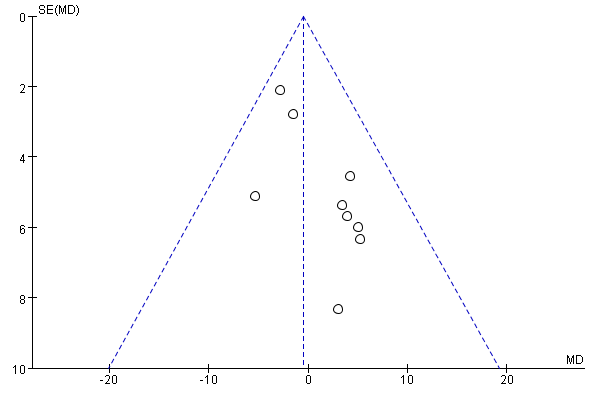


**A**


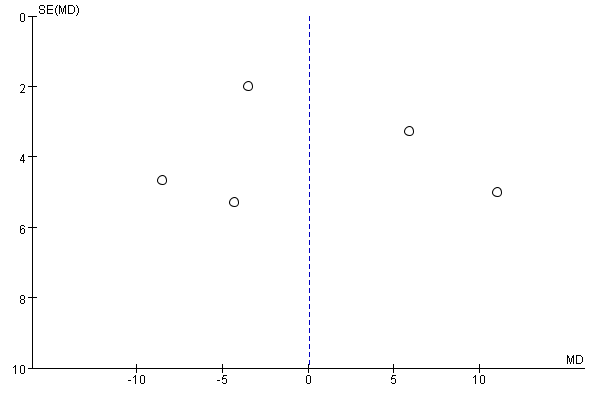


**B**


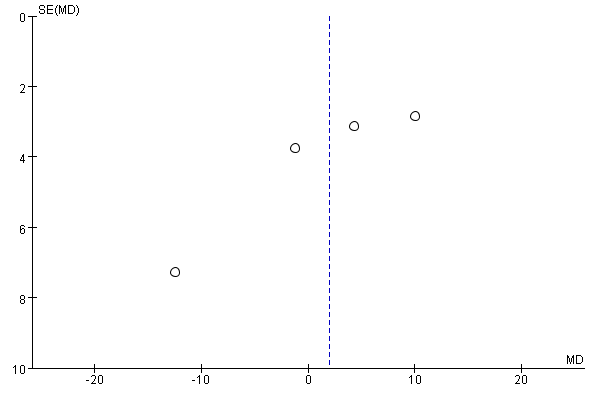


**C**


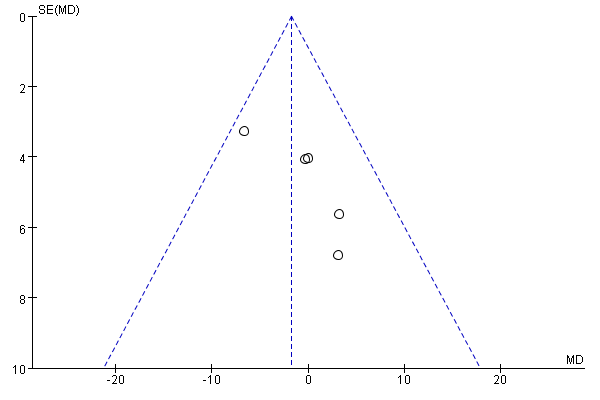


**D**


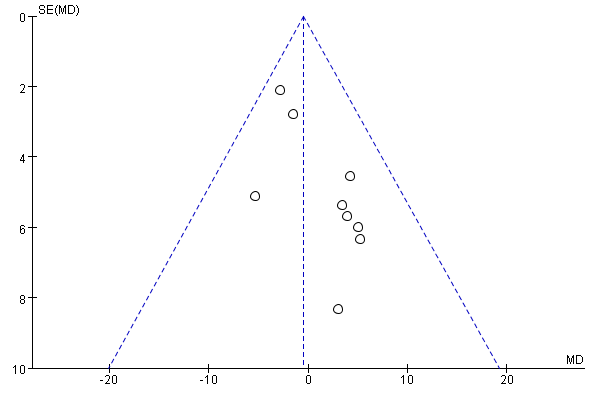


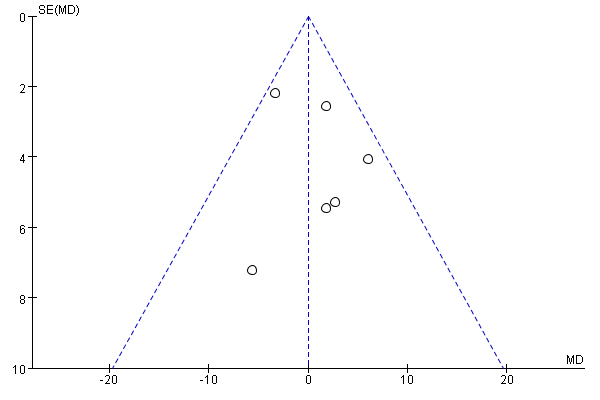
**E**

**F**
